# The antimicrobial peptide cathelicidin drives development of experimental autoimmune encephalomyelitis in mice by affecting Th17 differentiation

**DOI:** 10.1371/journal.pbio.3001554

**Published:** 2022-08-26

**Authors:** Katie J. Smith, Danielle Minns, Brian J. McHugh, Rebecca K. Holloway, Richard O’Connor, Anna Williams, Lauren Melrose, Rhoanne McPherson, Veronique E. Miron, Donald J. Davidson, Emily Gwyer Findlay

**Affiliations:** 1 Centre for Inflammation Research, University of Edinburgh, Edinburgh, United Kingdom; 2 Centre for Reproductive Health, University of Edinburgh, Edinburgh, United Kingdom; 3 United Kingdom Dementia Research Institute at The University of Edinburgh, Centre for Discovery Brain Sciences, Chancellor’s Building, The University of Edinburgh, Edinburgh, United Kingdom; 4 Centre for Regenerative Medicine, Institute for Regeneration and Repair, University of Edinburgh, Edinburgh Bioquarter, Edinburgh, United Kingdom; Tokyo Medical and Dental University Medical Research Institute, JAPAN

## Abstract

Multiple sclerosis (MS) is a highly prevalent demyelinating autoimmune condition; the mechanisms regulating its severity and progression are unclear. The IL-17-producing Th17 subset of T cells has been widely implicated in MS and in the mouse model, experimental autoimmune encephalomyelitis (EAE). However, the differentiation and regulation of Th17 cells during EAE remain incompletely understood. Although evidence is mounting that the antimicrobial peptide cathelicidin profoundly affects early T cell differentiation, no studies have looked at its role in longer-term T cell responses. Now, we report that cathelicidin drives severe EAE disease. It is released from neutrophils, microglia, and endothelial cells throughout disease; its interaction with T cells potentiates Th17 differentiation in lymph nodes and Th17 to exTh17 plasticity and IFN-γ production in the spinal cord. As a consequence, mice lacking cathelicidin are protected from severe EAE. In addition, we show that cathelicidin is produced by the same cell types in the active brain lesions in human MS disease. We propose that cathelicidin exposure results in highly activated, cytokine-producing T cells, which drive autoimmunity; this is a mechanism through which neutrophils amplify inflammation in the central nervous system.

## Abstract 2

A study of role of the antimicrobial peptide cathelicidin in autoimmunity shows that it is expressed in lesions of patients with multiple sclerosis and in the spinal cords of mice with experimental autoimmune encephalomyelitis; cathelicidin exposure promotes T cell activation and differentiation, and strongly induces interferon-gamma production in the inflamed nervous system.

## Introduction

In this project, we examined the differentiation of cytokine-producing T cells during multiple sclerosis (MS). MS is a demyelinating, neurodegenerative disease of the central nervous system (CNS) [1]. Through the modelling of MS using experimental autoimmune encephalomyelitis (EAE), we now understand that T cells play a central role in driving this disease [2–8]. However, the priming of pathogenic T cells during MS is complex and is still not fully understood. In particular, how other innate and adaptive immune cells affect longer-term T cell function in the CNS is unclear.

Like in other autoinflammatory conditions [9], the Th17 subset of cells is particularly important for driving disease in MS and EAE [10–14], through their ability to cross into the CNS following priming in the lymph nodes [15–18]; once there, Th17 cells also drive disease through contributing to blood–brain barrier (BBB) breakdown by attracting MMP-releasing neutrophils to the site [19–22].

Neutrophils have a dynamic relationship with T cells and it is well understood that they can influence T cell activation and migration [23,24]. There is now substantial evidence that neutrophils are important and pathogenic in EAE and MS. Neutrophil populations expand during both diseases and move into the CNS [25–27]. The peripheral neutrophil populations are also dysregulated, with an activated phenotype [28]. Depletion of neutrophils abrogates EAE disease [4,29,30]. The precise mechanisms through which neutrophils worsen EAE and increase severity of autoimmune conditions have not, however, been described; and while Th17 cell impact on neutrophils in the CNS is known [4], the reverse—the impact of neutrophils on Th17 cell differentiation and survival—is still unclear.

We have previously shown that neutrophil release of the antimicrobial host defence peptide cathelicidin, which occurs during degranulation and release of extracellular traps, potentiates Th17 differentiation in vitro and in models of acute inflammation [31]. However, its role in longer-term inflammation or in inflammation of the CNS is not known.

We now demonstrate that cathelicidin is not expressed in the healthy murine CNS or secondary lymphoid organs but is strongly produced during EAE and in the active demyelinated brain lesions of patients with MS. Cathelicidin production plays a fundamental role in disease pathogenesis. Mice lacking the peptide have reduced incidence of EAE as T cell production of proinflammatory cytokines is reduced. We propose that cathelicidin is critical for the development of pathogenic longer-term Th17 responses in inflammatory disease.

## Results

### Cathelicidin is expressed by multiple cell types in lymphoid organs and the central nervous system

Cathelicidin is released in lymph nodes during acute inflammation, yet whether this occurs during longer-term sterile inflammation is unknown. To assess this, we used the chronic inflammatory model, EAE, a model of immune-mediated demyelination in MS. We induced disease with the myelin oligodendrocyte glycoprotein (MOG) peptide MOG_35–55_ in wild-type (WT) C57BL/6JOlaHsD mice, following the standard protocol, and tracked signs of disease. Mice showed consistent onset of disease (S1A Fig) with mean day of symptom presentation being day 12 and median score being 2 (complete lack of tail mobility, altered gait and loss of balance). Inflammation and immune cell infiltration in the spinal cord increased consistently (S1B Fig), and T cells were detected in the spinal cord by immunohistochemistry from day 7 post-immunisation. The numbers of the total T cell population (S1C and S1D Fig) and IL-17 producers (S1E Fig) peaked at day 14, which is also the peak of symptoms, in agreement with previously published data [13,32].

As we hypothesised that exposure of these T cells to cathelicidin would promote their differentiation into Th17 cells and increase their pathogenicity, the first step was to establish whether and when cathelicidin exposure occurred, as it has not previously been characterised throughout a chronic inflammatory in vivo model. We therefore examined the inguinal lymph node, which drains the injection site, and the spinal cord, the site of inflammation.

**Fig 1:**
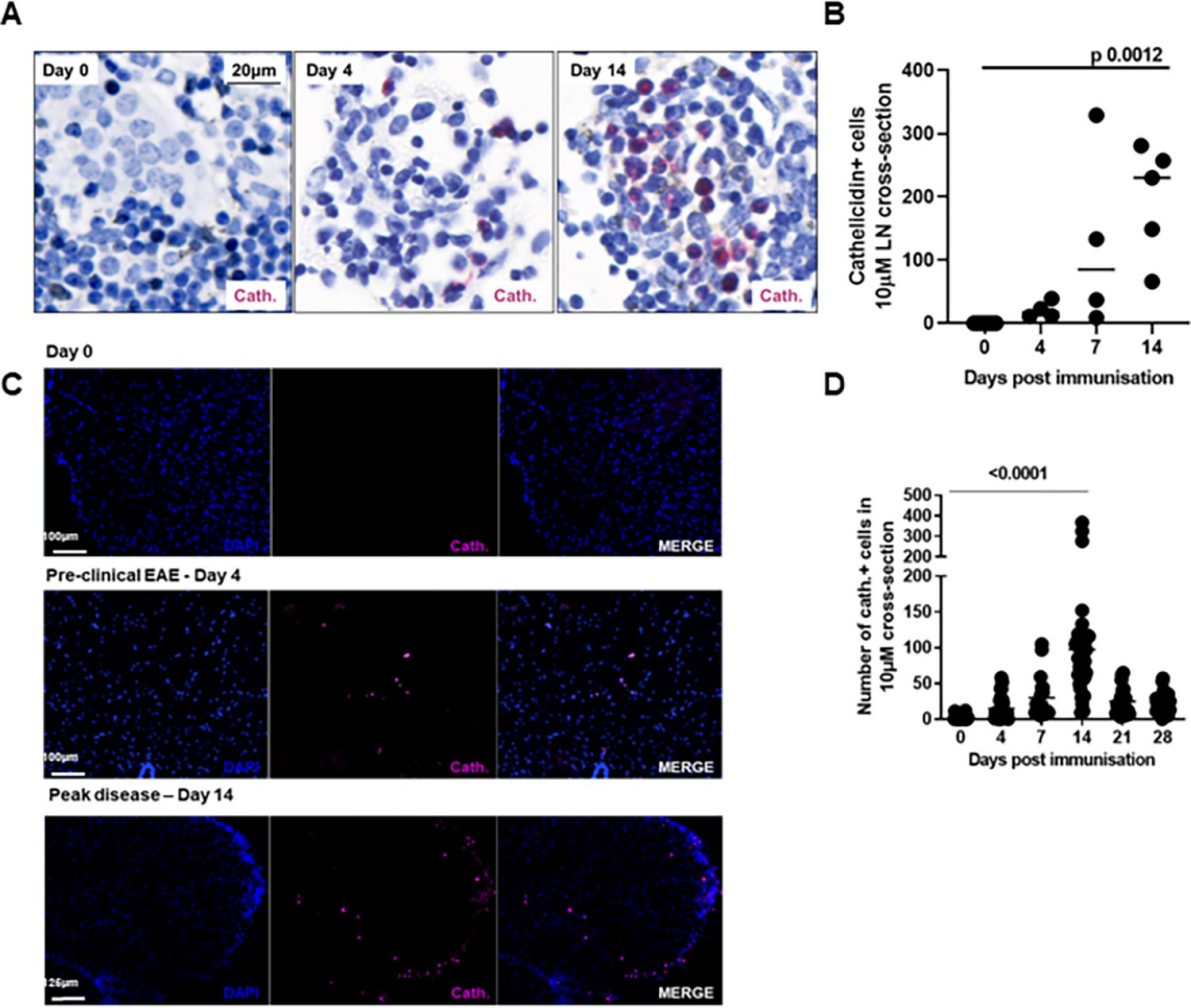
The antimicrobial peptide cathelicidin is produced in the CNS during EAE. EAE was induced in WT C57BL/6J mice on day 0 and mice observed for 28 days. Throughout the experiment, mice were humanely culled, perfused with 4% paraformaldehyde and cathelicidin-producing cells were quantified in (A, B) draining inguinal lymph nodes and (C, D) spinal cords. Statistical tests used: B—one-way ANOVA, D–two-tailed *t* test comparing day 14 to day 0. N values: A–images representative of 5–8 sections from 3 mice; B—3–5 sections from 3 mice; C–images representative of 3–4 mice; D– 13–42 sections analysed from 4–6 mice. Data available at 10.6084/m9.figshare.20310363. Cath, cathelicidin; CNS, central nervous system; EAE, experimental autoimmune encephalomyelitis; LN, lymph node; WT, wild-type.

In steady state mice, cathelicidin was not detected in lymph nodes (Fig 1A and 1B) or spinal cord (Fig 1C and 1D). However, as the immune response to immunisation developed, increasing amounts of cathelicidin were detected. We noted low-level detection in the inguinal lymph nodes as early as day 4 post-immunisation and it increased with time, with high levels of detection on day 14 (peak of disease) (Fig 1B).

In the spinal cord, cathelicidin was detected from day 4 post-immunisation, consistent with previous observations that neutrophils pass into the CNS very early after immunisation [4,25,30,33]. While cathelicidin-positive cells appeared during the preclinical stage of the disease, they were substantially increased during the acute phase (Fig 1C and 1D).

**Fig 2:**
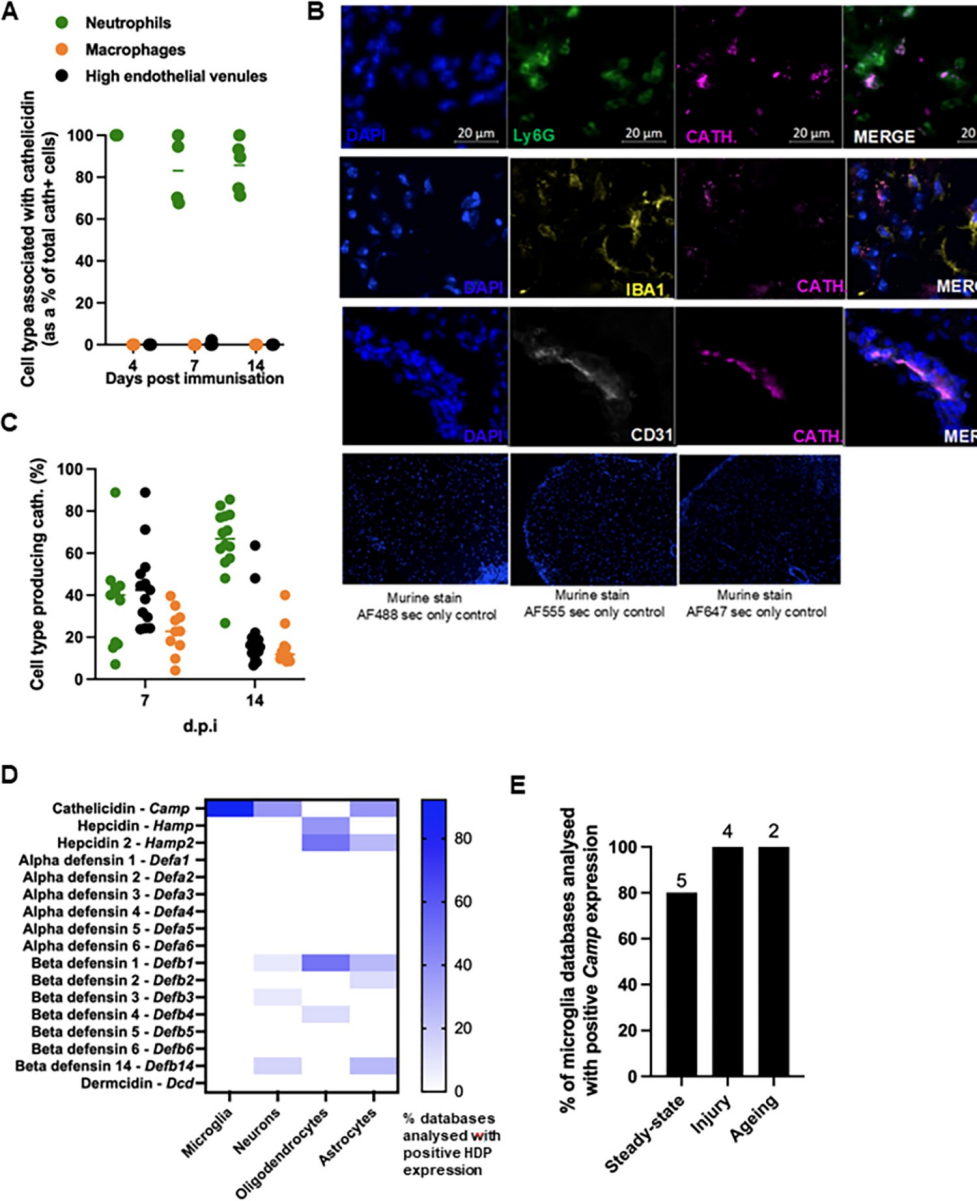
Cathelicidin is produced by multiple cell types in the CNS during EAE. EAE was induced in WT C57BL/6J mice on day 0 and mice observed for 28 days. Throughout the experiment, mice were humanely culled, perfused with 4% paraformaldehyde and cathelicidin-producing cells were quantified. On days 7 and 14 post-immunisation, co-staining was performed to determine production of cathelicidin by Ly6G+ neutrophils, F480^+^/Iba1^+^ microglia/macrophages, and CD31^+^ endothelial cells. (A) Shows the cell type associated with cathelicidin in the draining inguinal lymph node. (B) Example images from spinal cord on day 14 and contribution of each cell type to overall cathelicidin production in the (C) spinal cord was determined on day 14 post-immunisation. Analysis of published sequencing databases of murine microglia, astrocytes, oligodendrocytes, and neurons were performed. Databases were either accessed from supplementary information of the relevant papers or searched for in NCBI GEO Datasets. The search terms were as follows: “cell type” AND “rna seq” AND “mouse” NOT “iPSC.” (D) All datasets during steady state were analysed for expression of HDPs. The heatmap shows the percentage of databases analysed that expressed the named HDP. Blue indicates a high percentage of databases had positive expression, and white indicates a low percentage of databases had positive HDP expression. (E) The percentage of microglia databases analysed that had positive *Camp* expression in different conditions—steady-state, injury, and ageing. The number above the bar represents the number of databases analysed. Data shown are individual data points with line at median. N values: A– 3–5 sections from 3 mice; B and C– 13–18 sections from 4 mice; D–microglia– 13, neurons– 16, oligodendrocytes– 8, and astrocytes– 8; E–steady state– 5, injury– 4, ageing– 2. (Dataset information listed in S1 and S2 Tables). Data available at 10.6084/m9.figshare.20310363. Cath, cathelicidin; CNS, central nervous system; dpi, days post immunisation; EAE, experimental autoimmune encephalomyelitis; HDP, host-defence peptide; sec, secondary; WT, wild-type.

Having established that cathelicidin is expressed in the spinal cord, we next sought to determine which cells were releasing it. Cathelicidin is known to be inducible in a variety of cell types. It is predominantly produced by neutrophils and released from the secondary granules by degranulation [34,35]; it is also present on extracellular traps [36]. However, it is also expressed by other cells, including T cells [37,38], adipocytes [39], epithelium [40–42], and mast cells [43] in mice and humans. In the inguinal lymph node, cathelicidin was produced almost entirely by neutrophils (Fig 2A) with evidence of some having been released, as it was not associated with cells. This agrees with our previous work demonstrating that cathelicidin in the lymph nodes, following inoculation with heat-killed *Salmonella typhimurium*, is released from neutrophils [31]. However, cathelicidin in the murine spinal cord was detected in a variety of cell types. The majority of cathelicidin was associated with Ly6G^+^ neutrophils (approximately 70% of expression was from neutrophils on day 14) (Fig 2B and 2C), but some expression was noted in CD31^+^ endothelial cells (approximately 15% of expression) and Iba1^+^ microglia/macrophages (approximately 15% of expression).

We were interested in confirming these data by examining mRNA expression in CNS cells; it is possible that the observed cathelicidin production in Fig 2B and 2C was a result of its release by other cells and subsequent uptake by the CNS-resident cells such as microglia. To examine whether CNS cells were actively transcribing *Camp*, we analysed a series of published gene expression datasets of murine neuron, astrocyte, oligodendrocyte, and microglia cells (all datasets listed in S1 and S2 Tables). We analysed these cell datasets for the presence of a wide range of HDP (Fig 2D) and found cathelicidin was the most expressed HDP in the CNS. It was found to be transcribed in 92.3% of microglial datasets analysed, 37.5% of neuronal datasets, and 37.5% of astrocyte datasets. In contrast, it was not found to be transcribed in oligodendrocytes. Other HDP were not widely expressed (Fig 2D). The microglia results were interesting and agreed with our observation that cathelicidin was produced by these cells during EAE. Further examination of the microglial datasets demonstrated expression of cathelicidin in steady state (80% of datasets), injury such as spinal cord ligation (100%), and in aged mice (100%) (Fig 2E), suggesting a low level of continuous production.

Together, these data demonstrate that cathelicidin is widely produced during EAE, by neutrophils, and by resident CNS cells and that it persists longer term over the course of disease, with expression peaking at maximal disease severity.

### Cathelicidin is expressed during human multiple sclerosis disease

**Fig 3:**
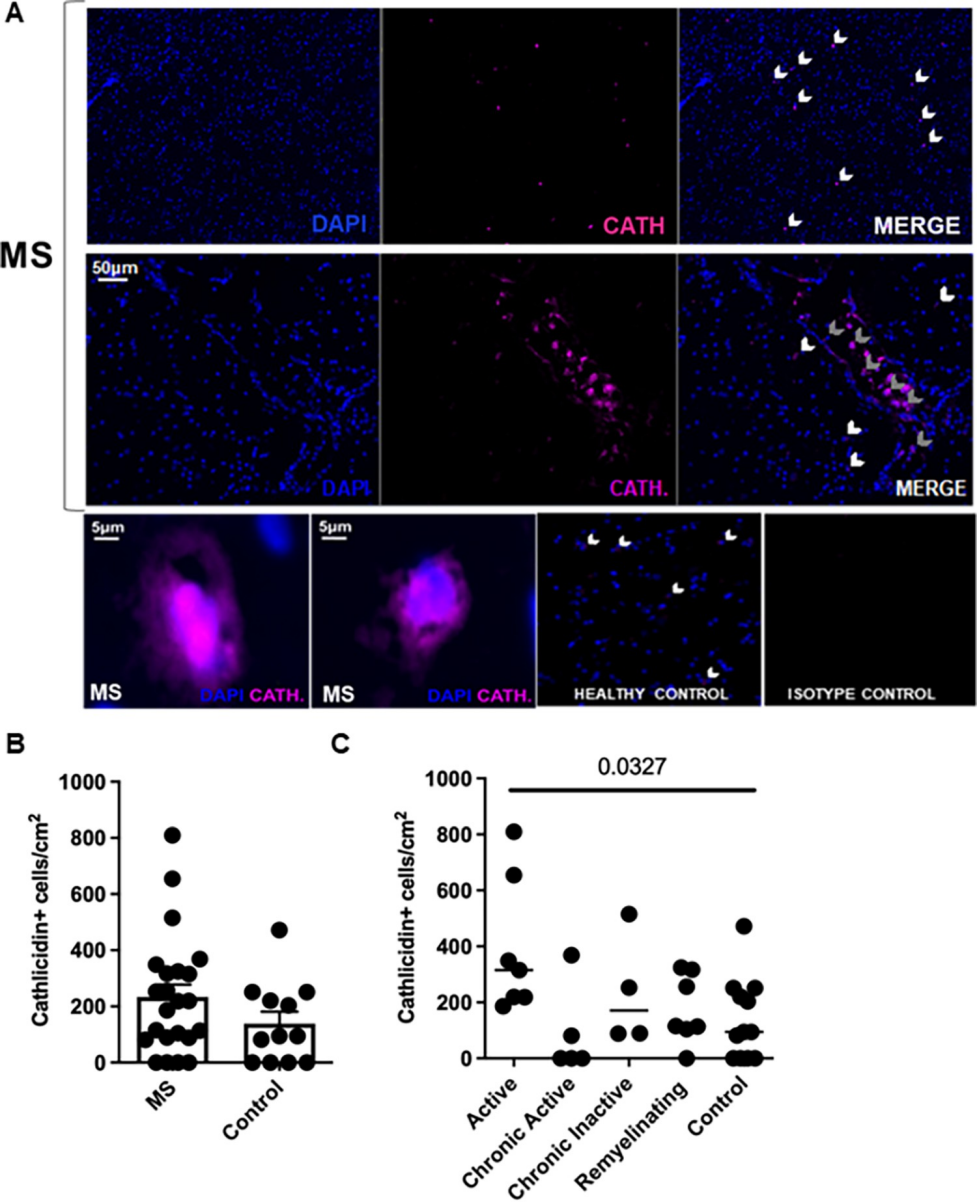
Cathelicidin is produced in the healthy and inflamed human brain. Postmortem brain sections were obtained from patients who died of MS and control patients with no neurological disease. (A-C) Cathelicidin (Cath.) production was quantified using immunofluorescent imaging. Grey arrowheads indicate cathelicidin within blood vessels, and white arrowheads indicate cathelicidin outside vessels. Data shown are (B) mean with error bars showing standard error and (C) individual data points with line at median. Statistical test used: in (C)–one way ANOVA. N values: B and C– 7 patients in MS group and 3 patients in control group, each data point represents an area of the brain analysed. Images in (A) are representative of at least 3 patients. Patient information is listed in S3 Table. Data available at 10.6084/m9.figshare.20310363. cath, cathelicidin; MS, multiple sclerosis.

Next, we wished to determine whether cathelicidin is also expressed in human MS. To do so, we analysed postmortem brain tissue from 7 patients from the UK Multiple Sclerosis Tissue Bank (patient information listed in S3 Table). Expression of cathelicidin was noted, surprisingly, in control brain tissue as well as in MS brain tissue (Fig 3A and 3B). We noted that this expression was localised and next quantified cathelicidin in separate lesions, using the International Classification of Neurological Diseases guidelines. Lesions develop from normal-appearing white matter. Active lesions are characterised by an influx of immune cells, activated resident microglia, and demyelination. Lesions may undergo active remyelination but can also become chronically inactive, a situation in which there is extensive demyelination and few immune cells present. Moreover, lesions can become chronically active with a heavily demyelinated core and activated glial cells at the rim [44–46]. We noted a significant increase in cathelicidin expression in the active demyelinated lesions (Fig 3C). In contrast, the chronic active, chronic inactive, and remyelinating lesions did not have increased cathelicidin compared to control tissue (Fig 3C).

**Fig 4:**
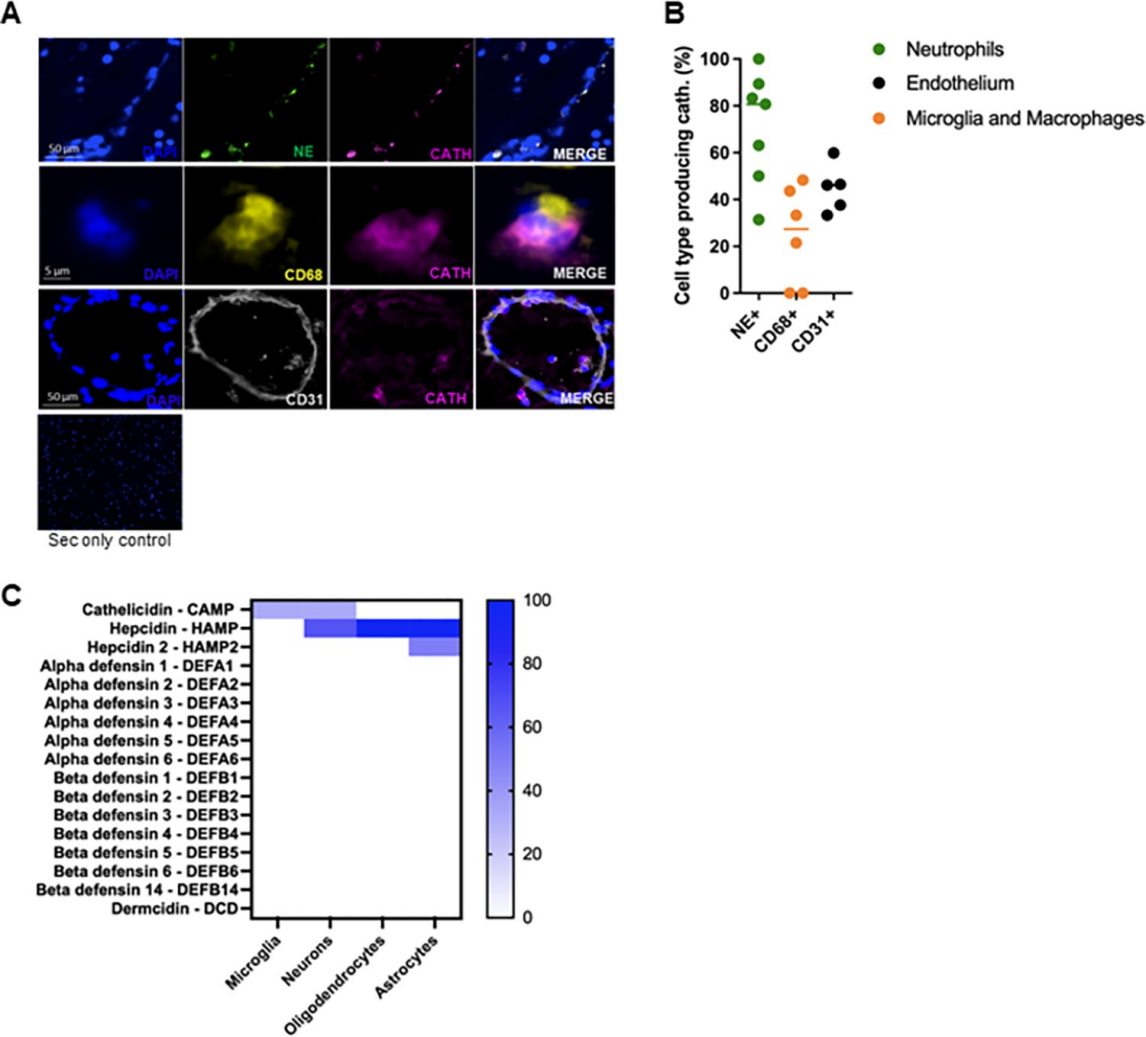
Cathelicidin is produced by multiple cell types in the human CNS. Postmortem brain sections were obtained from patients who died of MS and control patients with no neurological disease. (A-C) Cathelicidin (Cath.) production was quantified using immunofluorescent imaging. (A) Co-staining was performed to determine production of cathelicidin by neutrophil elastase (NE)+ neutrophils, CD68+ microglia/macrophages, or CD31+ endothelial cells. (B) Contribution of each cell type to overall cathelicidin production was determined in the active lesions. (C) Published RNA sequencing human databases were analysed for HDP expression. Databases were either accessed from supplementary information of the relevant papers or searched for in NCBI GEO Datasets. The search terms were: “cell type” AND “rna seq” AND “human” NOT “iPSC.” A heatmap showing HDP expression as a percentage of databases analysed is shown. Data shown in B are individual data points with line at median. N values: A and B– 7 patients in MS group and 3 patients in control group, each data point represents an area of the brain analysed; C– 3 datasets for neurons, 2 for astrocytes, 5 for microglia, and 1 for oligodendrocytes (dataset information listed in S4 Table). Data available at 10.6084/m9.figshare.20310363. cath, cathelicidin; CNS, central nervous system; HDP, host-defence peptide; MS, multiple sclerosis; NE, neutrophil elastase; sec, secondary.

In the active lesions, the same cell types co-localised with human cathelicidin as we had observed in EAE—namely neutrophils (neutrophil elastase, NE+), microglia/macrophages (CD68+), and endothelial cells (CD31+) (Fig 4A and 4B). Quantification of active lesions was performed to identify the proportion of cathelicidin contributed by each cell type; the pattern in these lesions was roughly similar to murine samples, with the majority of the cathelicidin coming from neutrophils and a substantial minority from endothelial cells and microglia/macrophages.

Again, we downloaded published RNA sequencing data from experiments performed on human CNS-resident cells (datasets used listed in S4 Table) in order to confirm that cathelicidin is produced by these cells and not simply taken up by them. We found that cathelicidin is produced by microglia and neurons, as in the murine datasets (Fig 4C) but not in oligodendrocytes or human astrocytes. In contrast to mice, cathelicidin was not the highest-expressed HDP in the human CNS, with hepcidin being widely produced as well. However, the pattern of expression noted in these datasets matched what we had observed at the protein level. Together, these data show that cathelicidin is expressed by a variety of cell types in the human and murine CNSs during chronic neuroinflammation.

### Cathelicidin promotes severe disease in EAE

**Fig 5:**
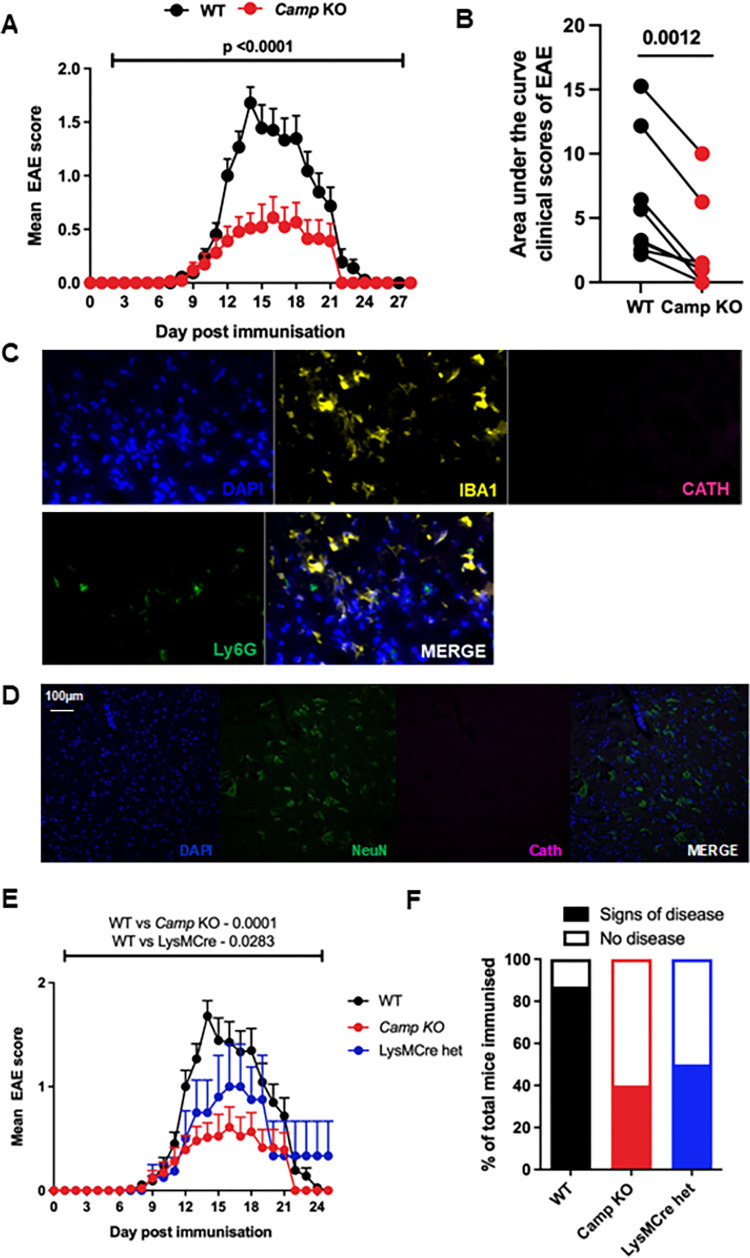
Mice lacking cathelicidin are resistant to EAE disease. EAE was induced in WT C57BL/6J, cathelicidin KO (*Camp KO*) mice and LysMCre conditional KO mice on day 0. (A) Clinical signs of illness were noted throughout the experiment and (B) area under the curve calculated for each individual experiment. (C-E) Conditional KO LysMCre mice lacking cathelicidin in myeloid-lineage cells were generated (see S2 Fig) and (C) immunofluorescence performed to confirm microglia and neutrophils lacked cathelicidin. (D) WT mice were culled on day 14 post-immunisation and spinal cord isolated. Spinal cord cross-sections were co-stained for neuronal marker NeuN (green) and cathelicidin (magenta), which showed that neurons did not co-localise with cathelicidin in the spinal cord. (E, F) Clinical signs of disease were tracked in conditional KO mice. Data shown are (A, E) mean and standard error, (B) individual EAE experiments. N values: A—WT 85, *Camp*^*−/−*^ 71; E—WT 85, *Camp*^*−/−*^ 71, LysMCre 8. Images in C are representative of 3 mice. Statistical tests used: A and E–one-way ANOVA, B–paired two-tailed *t* test. Data available at 10.6084/m9.figshare.20310363. Cath, cathelicidin; EAE, experimental autoimmune encephalomyelitis; het, heterozygous; KO, knockout; WT, wild-type.

Next, we examined whether expression of cathelicidin is required for disease, by inducing EAE in mice lacking cathelicidin (*Camp*^*−/−*^, knockout, KO), first produced in [47]. Observation of clinical signs of illness demonstrated that KO mice consistently showed significantly attenuated disease compared to WT mice (Fig 5A and 5B).

Give the multiple cell types known to be capable of expressing cathelicidin, particularly in the context of inflammation, we wanted to start to define the key cellular compartment for cathelicidin production affecting EAE disease severity, so generated a new conditional cathelicidin KO mouse. This LysMCre mouse lacks cathelicidin in myeloid-lineage cells (generation described in S2 Fig). This mouse had no cathelicidin in microglia or neutrophils (Fig 5C). LysM can also be observed in neurons [48], but no cathelicidin was seen to be produced by WT neuronal cells in our system (Fig 5D). Observation of clinical signs of illness over time (Fig 5E) and incidence of disease (Fig 5F) showed the conditional KO mice phenocopied the full KO animals and had attenuated incidence of disease, demonstrating that myeloid cell production of cathelicidin is sufficient for full disease penetrance and therefore that the noted endothelial cell production is not essential for development of disease.

### Cathelicidin does not affect cell infiltration to the spinal cord but increases T cell activation

**Fig 6:**
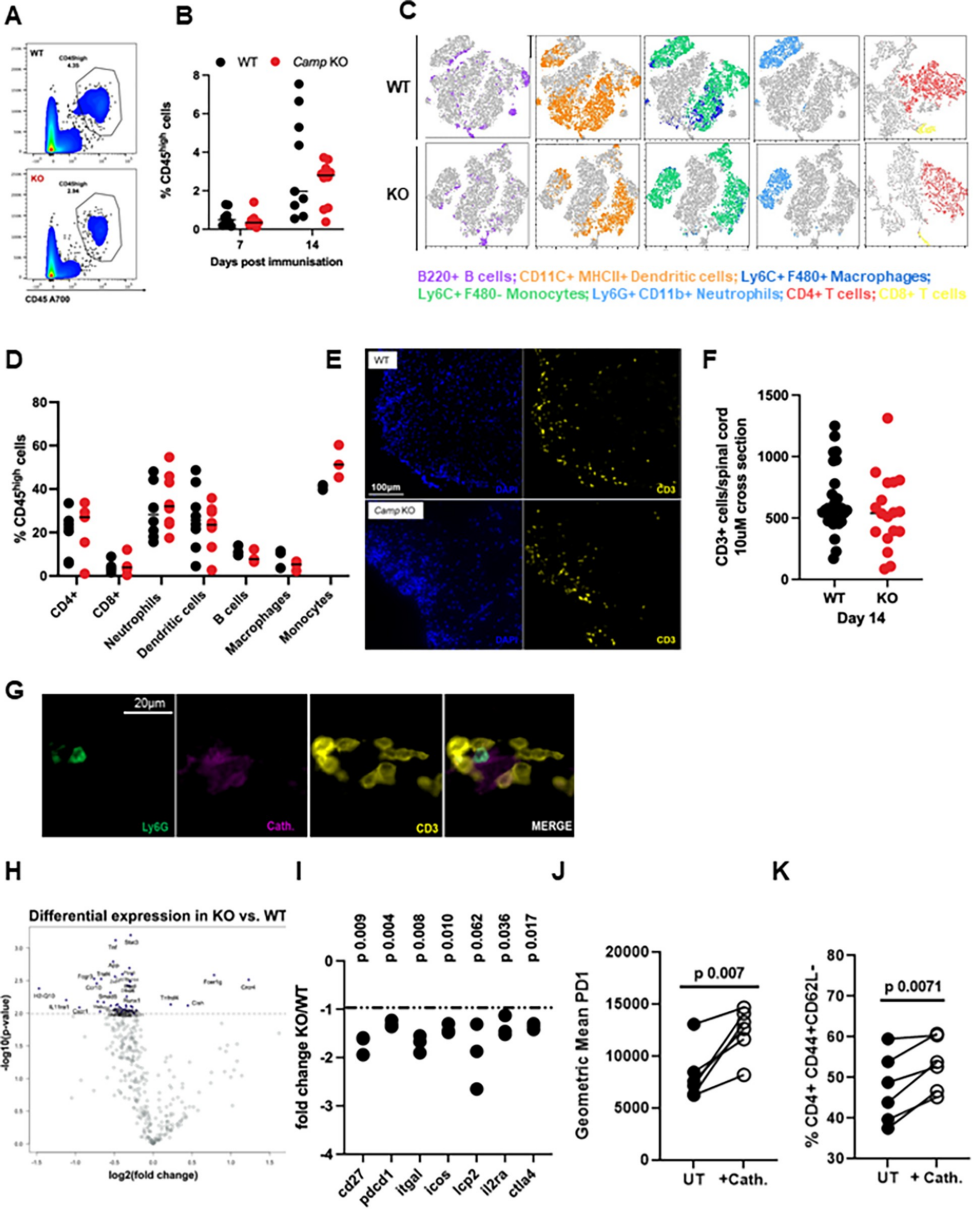
Mice lacking cathelicidin are resistant to induction of EAE despite normal infiltration of spinal cord T cells. EAE was induced in WT C57BL/6J and cathelicidin KO (*Camp−/−*, KO) mice on day 0. (A, B) On days 7 and 14, CD45^high^ cell infiltration into the spinal cord was quantified by flow cytometry. (C, D) Immune cell subsets in the CD45^high^ gate were delineated on day 14 in the spinal cord then (E, F) CD3^+^ T cell numbers in the spinal cord were confirmed on day 14 by immunofluorescence. (G) Neutrophil release of cathelicidin was observed in close contact with T cells in the spinal cord. (H, I) On day 7 draining inguinal lymph nodes were removed and CD4^+^ T cells isolated. Gene expression differences between WT and KO T cells were assessed on a Nanostring mouse immunology chip (J, K) Splenic CD4^+^ T cells from naïve WT mice were incubated with CD3/CD28 stimulation and 2.5 μM synthetic cathelicidin for 24 hours before activation was assessed by flow cytometry. Data shown are individual data points with (B, D, F) line at median. Statistical tests used: (I) two-tailed *t* tests with multiple comparison correction; (J, K) paired two-tailed *t* test. N values: A and B– 8–10; C–representative plot of 3–9 mice; D– 3–8; F– 18–29 sections from 3–4 mice; I– 3, J– 6, K– 6. Data available at 10.6084/m9.figshare.20310363. Cath, cathelicidin; EAE, experimental autoimmune encephalomyelitis; KO, knockout; UT, untreated; WT, wild-type.

Having shown that cathelicidin is important for EAE development, we next examined its mechanism of action. We have previously demonstrated that cathelicidin induced survival of T cell subsets [31] and others have shown it to be a chemoattractant for T cells [49]. As T cells drive pathology in EAE [16,18,50–53], we therefore hypothesised that fewer T cells would be present in the spinal cord of KO mice and that this was the cause of the reduced disease severity. To test this, we carried out a full flow cytometric phenotyping of spinal cord immune cells over time in both WT and KO mice (gating strategy shown in S3 Fig). To our surprise, there were no observable differences in leukocyte infiltration—including T cell subsets—between WT and KO mice during the EAE timecourse (Fig 6A and 6B). In addition, the relative proportions of different subsets of immune cells detected in the spinal cord were not significantly different between the genotypes (Fig 6C and 6D). To further confirm these findings, we performed immunofluorescent staining for CD3^+^ T cells in WT and KO spinal cord and found no differences in number or location on day 14 post-immunisation (Fig 6E and 6F).

Together, these data show that cathelicidin is widely expressed during EAE and plays an important, nonredundant role in disease pathogenesis. However, this was not secondary to a decrease in the total number of T cells or other immune cells entering the CNS.

As the overall magnitude of immune cell flux into the CNS was similar in WT and KO mice, and spinal cord T cell numbers at the peak of disease were the same, we therefore hypothesised that the phenotype of the T cells, and the cytokines they produced, were altered in the absence of cathelicidin. Production of proinflammatory cytokines from T cells is of critical importance to the development of severe EAE disease [18,54–56]. We have previously shown that cathelicidin strongly preferentially potentiates Th17 but not Th1 or Th2 differentiation during acute responses [31] and so we hypothesised the same phenomenon was occurring during EAE, particularly as we observed that T cells in the lymph nodes and spinal cord (Fig 6G) were exposed to cathelicidin during disease.

To examine this, we decided to characterise T cells in the draining lymph node, just before they move into the spinal cord and initiate observable disease. We isolated CD4^+^ T cells from the inguinal node of WT and KO mice on day 7 post-immunisation and analysed gene expression differences between the strains of mice, using a Nanostring Immunology chip. A large number of genes were altered between WT and KO T cells (Fig 6H).

Firstly, T cells isolated from the inguinal lymph nodes of KO mice (therefore differentiating in the absence of cathelicidin after induction of EAE) were less activated than those from WT mice. In naïve mice, activation status of or cytokine production by T cells in KO mice is not different to WT mice (S4 Fig; [31]). However, in EAE expression of genes encoding CD27, PD1, LFA-1, ICOS, and LCP2 were significantly lower in KO CD4^+^ T cells (Fig 6I). To confirm these findings at the protein level and to examine if this was a direct effect of cell exposure to cathelicidin, we incubated isolated splenic CD4^+^ T cells with synthetic cathelicidin ex vivo, alongside CD3/CD28 stimulation. In this experiment we observed, agreeing with the Nanostring data, a significant up-regulation of PD1 (Fig 6J) and CD44 expression, and a loss of CD62L (Fig 6K) following exposure to cathelicidin. These data suggest that cathelicidin is able to increase T cell activation.

### Cathelicidin drives Th17 cell differentiation and plasticity

**Fig 7:**
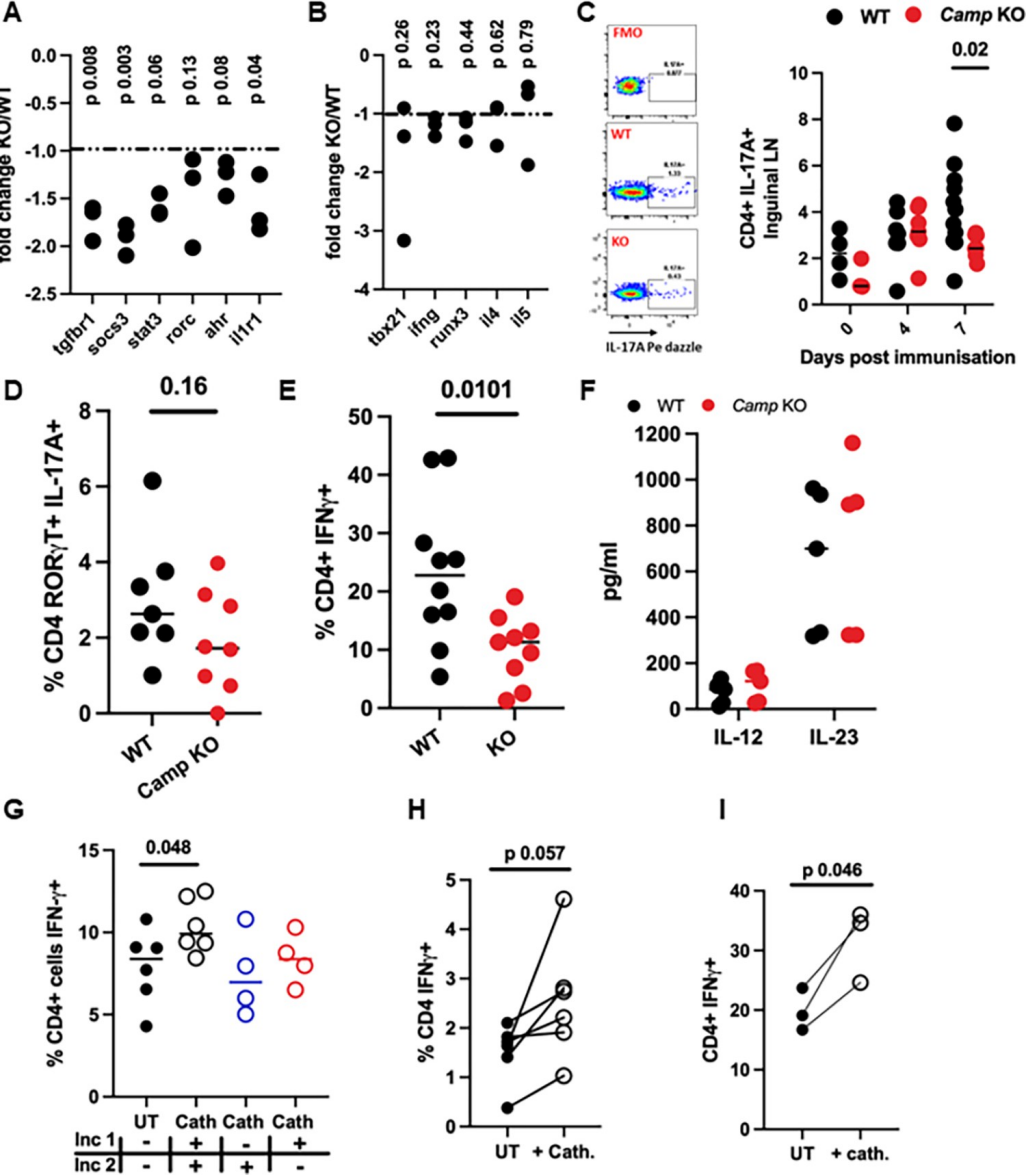
In the absence of cathelicidin T cell differentiation into Th17 cells and to exTh17 cells is impaired. EAE was induced in WT C57BL/6J and cathelicidin KO (*Camp−/−*, KO) mice on day 0. On day 7, draining inguinal lymph nodes were removed and CD4^+^ T cells isolated. (A-B) Gene expression differences between WT and KO T cells were assessed on a Nanostring mouse immunology chip, and (C) on days 0, 4, and 7, IL-17 production was assessed by flow cytometry in the inguinal lymph nodes. (D, E) On day 14, cytokine production was assessed by flow cytometry or (F) by ELISA in the spinal cord. (G) WT splenic CD4^+^ T cells were cultured with Th17 driving medium for 48 hours (incubation 1, inc 1) then recombinant IL-12 for 72 hours (incubation 2, inc 2) before cytokine production was assessed by flow cytometry. (H) On day 7 of EAE inguinal lymph nodes were removed from WT mice and incubated for 72 hours with IL-12 and synthetic cathelicidin. (I) Human peripheral blood T cells were incubated with TGF-β and anti-CD3/CD28 stimulation then with IL-12 for 3 days, in the presence or absence of recombinant human cathelicidin (LL-37), after which IFN-γ was measured by flow cytometry. Data shown are individual data points with lines at median. N values: A, B– 3; C– 3–11; D– 7–8; E– 9–10; F– 5; G– 6 for UT and Cath ++, 4 for Cath −+ and +−; H– 6, I–3. Statistical tests used: (A, B)–two-tailed *t* tests with post hoc correction for multiple comparisons; (C)–two-tailed *t* test; (D, E)–two-tailed *t* test; (G, H, I)–paired two-tailed *t* test. Data available at 10.6084/m9.figshare.20310363. Cath, cathelicidin; EAE, experimental autoimmune encephalomyelitis; inc, incubation; KO, knockout; UT, untreated; WT, wild-type.

Next, we noted that many genes encoding proteins that are part of the Th17 differentiation pathway were down-regulated in KO mice. These included the TGF-β receptors, STAT3, RORγt, and the aryl hydrocarbon receptor AHR, and SOCS3 (Fig 7A). The large number of genes modulated by the absence of cathelicidin suggest this is a key pathway through which cathelicidin mediates its functional impact, supporting our previous work in this area. Importantly, showing specificity in the immunomodulation, genes relating to the Th1 and Th2 pathways were not altered in KO mice (Fig 7B), demonstrating in vivo that cathelicidin does not suppress all cytokine production indiscriminately but specifically affects Th17-related genes.

To examine this at the protein level, we isolated CD4^+^ T cells from the inguinal node. The production of IL-17A was indeed significantly suppressed in mice lacking cathelicidin compared to WT animals at day 7 in the disease course (Fig 7C), but not at baseline, indicating that KO mice do not demonstrate this defect prior to an immunological challenge. CD4^+^ T cells established a basal level of IL-17A production in response to EAE induction, regardless of genotype (Fig 7C); however, when the Th17-biased immune response amplified at day 7 in WT mice, the same amplification did not occur in KO mice. This was associated with protection from severe pathology, in concordance with multiple previous studies demonstrating that a reduction in IL-17 production leads to less severe EAE disease [12,13,57].

IL-17-producing Th17 cells are, however, not the whole story in EAE pathogenesis. There is considerable plasticity of Th17 subsets depending on the cytokines to which they are exposed [11,58–63]. In particular, Th17 cells (RORγt^+^, producing IL-17), which are exposed to IL-12 or to IL-23, up-regulate IFN-γ production in a STAT4- and Tbet-dependent fashion [58,59]. Therefore, during EAE, early RORγt^+^ IL-17A^+^ cell populations in the draining lymph node lose IL-17A and become a pathogenic IFN-γ^+^ “ex-Th17” population [11,61–65]—indeed, almost all the T cell IFN-γ production in the spinal cord is from cells that were once IL-17 producers [11]. Early suboptimal Th17 potentiation in the lymph node, in the absence of cathelicidin, could modulate this process, with a consequent lessening of the frequency of these cells in the CNS. We hypothesised that cathelicidin potentiates EAE pathology not only by enhancing early Th17 differentiation but also by influencing later Th17 to exTh17 conversion.

To test this, we looked at cytokine production in the spinal cord on day 14 of EAE. Th17 cell numbers in the spinal cord were reduced at peak disease, but not significantly (Fig 7D). Instead, in agreement with our hypothesis, we noted a significant reduction in IFN-γ production by CNS CD4^+^ T cells (Fig 7E).

The conversion of Th17 cells to IFN-γ producers occurs following IL-12 or IL-23 signals [58,59,64,66]. It was possible that cathelicidin KO mice had less of these cytokines in the CNS, but we confirmed that this was not the case with spinal cord wash ELISAs (Fig 7F). Therefore, we proposed that cathelicidin is able to potentiate the response to the available CNS cytokines in Th17 cells in the same way it potentiates responses to TGF-β in naive T cells.

To investigate this, we cultured WT splenic T cells in Th17 differentiation medium for 48 hours in the presence or absence of 2.5 μM cathelicidin. Cells were then washed and reincubated with IL-12 to promote IFN-γ production, again in the presence or absence of cathelicidin. We found (Fig 7G) that cathelicidin strongly promoted IFN-γ production in cells that had previously been in Th17-driving conditions.

We wondered at which point cathelicidin signals are important for this conversion to IFN-γ producers. To answer this, 2 stages of cathelicidin exposure were provided—either in the initial Th17-driving conditions or in the later IL-12 medium (Fig 7G). We found that if cathelicidin was included in one of these stages, no boosting of IFN-γ production was seen—but if it was included in both stages, significant enhancement of IFN-γ occurred. This suggests that the full range of cathelicidin-producing cells described in Figs 1 and 2 may contribute to this phenotype, with neutrophils initially promoting Th17 differentiation in the lymph nodes and priming for transition to ex-Th17 cells, but also spinal cord neutrophils and microglia/macrophages potentially driving this pathogenic T cell plasticity at the site of damage.

Next, we investigated whether T cells in the draining lymph nodes of mice during early EAE would enhance their IFN-γ production if they were exposed to cathelicidin ex vivo. We immunised WT mice and removed the draining inguinal lymph nodes on day 7 post-immunisation. We restimulated lymph node single cell suspensions with MOG peptide in the presence of IL-12 to induce conversion of antigen-specific T cells (Fig 7H). Exogenous cathelicidin added in to this process enhanced IFN-γ production, indicating that exposure to cathelicidin in the spinal cord would be expected to further enhance this conversion to ex-Th17 cells.

Finally, we determined whether human T cells respond in the same way as murine T cells to cathelicidin. We cultured peripheral blood T cells from healthy adult human donors with TGF-β and then with IL-12, in the presence or absence of 2.5 μM human cathelicidin (LL-37). Cells that were exposed to cathelicidin produced significantly more IFN-γ in these conditions than those that did not (Fig 7I). This therefore agrees with the murine data.

Together, these data indicate that the generation of a pathogenic ex-Th17 population of T cells in the CNS is impaired in the absence of cathelicidin signalling. These results lead us to propose a model in which cathelicidin has 3 impacts on the generation of pathogenic T cells in EAE. Firstly, it directly increases the activation of T cells. Next, it specifically potentiates Th17 differentiation and IL-17 production in the lymph node. Finally, it enhances Th17 to exTh17 differentiation and IFN-γ production in the spinal cord.

## Discussion

Here, we demonstrate that the antimicrobial peptide cathelicidin drives severe autoimmune disease in the mouse model of MS. Specifically, exposure of CD4^+^ T cells in the draining lymph node to neutrophil-derived cathelicidin enhances their differentiation into Th17 cells. When these cells move into the CNS, they are further exposed to cathelicidin released by neutrophils, microglia, and endothelial cells, and this potentiates Th17 differentiation into IFN-γ producing “exTh17” cells. This work extends previous studies showing that neutrophils are important in autoimmune disease by providing a mechanism through which they can drive inflammation—the specific potentiation of IFN-γ-producing exTh17 cells in the CNS.

Cathelicidin is widely expressed in autoimmune and autoinflammatory conditions [67] as well as during infections and plays a key role in driving inflammation in these diseases—including chronic obstructive pulmonary disease [68], psoriasis [69,70], and atherosclerosis [71]. A number of proinflammatory mechanisms of action of cathelicidin have been described including activation of the NLRP3 inflammasome [72], promoting uptake of self-nucleic acids [36,67,69], and enhancement of cytokine and chemokine release [43,73–75]. To these, we now add specific promotion of pathogenic T cell differentiation and long-term influence on adaptive T cell cytokine production. We have previously shown that cathelicidin induces Th17 cell differentiation through potentiation of TGF-β and aryl hydrocarbon receptor signalling—these are pathogenic cells that induce disease in a number of autoimmune conditions [2,9,76,77]. Previously, cathelicidin has been shown to be recognised as a T cell autoantigen in psoriasis [78] and to chemoattract T cells in culture [49], suggesting multiple mechanisms through which the peptide could alter autoimmune T cell development and function. Indirect mechanisms are also possible, particularly through an impact on dendritic cell function. We have shown that cathelicidin alters dendritic cell differentiation and priming of T cells [79,80], and others have shown it can form immune complexes with DNA to promote plasmacytoid DC activation [69].

This means that the impact of this innate peptide is likely to be wide-ranging in a number of autoimmune diseases driven by T cell dysfunction. As well as psoriasis and MS, this is therefore likely to be important in diseases such as neuromyelitis optica and Crohn’s disease, both of which feature pathogenic Th17 cell populations and large influxes of neutrophils into inflamed tissues [81–86].

We demonstrate here that cathelicidin is expressed in the healthy human brain. The purpose of this cathelicidin release is currently unknown. We also demonstrated that cathelicidin was widely released in the inflamed mouse spinal cord during EAE and active MS lesions in human brain. It has not previously been described in MS or EAE, although expression has been noted in the olfactory bulb, cerebellum, medulla oblongata, and spinal cord during experimental meningitis expression [87]. The expression of cathelicidin in our study agrees with previous work showing its release in other systems can be triggered by mediators released during sterile inflammation—such as leukotriene B4 [88] or phenylbutyrate [89]—as well as strictly infection-triggered mediators. A greater knowledge of the role of antimicrobial peptides in sterile inflammation and in the CNS in particular is required as we now begin to understand the wide-ranging effects these peptides have on the immune system.

We observed co-localisation of cathelicidin with microglia and endothelial cells as well as neutrophils. It is difficult to rule out co-localisation owing to nearby neutrophil degranulation releasing cathelicidin and it being taken up by other cells. We believe that microglia are actively producing cathelicidin as our mining of published datasets revealed microglia can express the cathelicidin gene. Also, there is supporting evidence from previous studies; endothelial production of cathelicidin has been noted in meningitis infections of mice [90], and microglial production has been demonstrated in vitro [91–93]. The question of which cells are important for releasing cathelicidin into the vicinity of CNS T cells remains open. We have also attempted to answer this question with our conditional KO lysMcre mice. EAE in these mice—which do not have cathelicidin in their microglia or neutrophils but do in their endothelial cells (and in other cells such as mast cells and epithelium)—has an almost identical disease course to full KO mice. This implies that the neutrophil cathelicidin in the lymph node and microglial cathelicidin in the spinal cord are key but that endothelial cathelicidin is unimportant. Relative contributions from microglia and neutrophils in the spinal cord are the focus of current research. In particular, it would be instructive to generate microglia-specific, macrophage-specific, and neutrophil-specific cathelicidin KO mice. This would allow us to unravel the impact of cathelicidin produced by each cell type and how each cell’s production differentially affects the incoming T cells.

The differentiation of Th17 cells in the draining inguinal lymph node and its enhancement by cathelicidin agrees with our previous data [31] where we demonstrated this phenomenon occurring in the 72 hours following inoculation with heat-killed pathogens and with influenza virus. Now, we extend those studies by demonstrating this neutrophil-driven Th17 potentiation occurs in longer-term inflammatory models.

The exTh17 conversion experiments performed ex vivo demonstrate that both early (lymph node) and late (CNS) cathelicidin are important for the final differentiation of IFN-γ-producing cells. Our finding here, that cathelicidin induces IFN-γ production in cells previously incubated in Th17-driving conditions, is surprising. In our previous studies, we found that cathelicidin incubated with naïve T cells does not affect IFN-γ production (positively or negatively) in Th1-driving conditions, and it suppresses IFN-γ in cells that are actively receiving TGF-β signals (both of which were published in [31]). These disparate outcomes on cytokine production in naïve and activated T cells, in different cytokine milieu, demonstrate the complexity of these systems. Unravelling the signalling pathways involved will be critical. Interestingly, the further differentiation of spinal cord Th17 cells into exTh17 cells [11,59] has been shown to be dependent on the aryl hydrocarbon receptor [11]. Our previous studies demonstrated that initial enhancement of Th17 cells by cathelicidin is likewise AhR-dependent, and we hypothesise that a potentiation of AhR signalling in the spinal cord is occurring in T cells, which come into contact with released cathelicidin there.

Recent studies have demonstrated the gut microbiome influences outcome in EAE (reviewed in [94]). We know that cathelicidin KO mice have an altered microbiome [95,96]; however, we do not believe that the gut microbiome has a critical importance in our studies owing to the fact that WT splenic T cells given cathelicidin in vitro behave the same as T cells in vivo in WT mice. In addition, naïve KO and WT mice have no differences in T cell activation or cytokine production. However, it may be relevant that our mice were housed in individually ventilated cages in SPF conditions, and mice housed differently may show altered gut and, subsequently, systemic immune responses.

Overall, this study describes antimicrobial peptide expression in the CNS and establishes the role of cathelicidin in directing pathogenic T cell responses in long-term inflammation. This work extends previous studies showing that neutrophils are important in autoimmune disease by providing a mechanism through which they can drive inflammation—the specific potentiation of IFN-γ-producing cells in the CNS.

## Methods

### Mice

WT C57Bl/6JOlaHsd and cathelicidin KO (*Camp*^*tm1Rig*^, KO) and *LysMCreFLCamp* mice were bred and housed in individually ventilated cages, under specific pathogen-free conditions. Female mice between 9 and 13 weeks of age were used for EAE experiments; for in vitro cell stimulations, both male and female mice between 6 and 12 weeks were used. We have previously determined no sex differences exist in Th17 potentiation by cathelicidin. KO mice were backcrossed onto WT stocks for 10 generations. Mice were housed in accordance with the ASPA code of practice for the UK; this includes temperatures from 19 to 24°C, humidity from 45% to 65%, and a 12-hour light/dark cycle.

All animal experiments were performed by fully trained personnel in accordance with Home Office UK project licences PAF438439 and 70/8884, under the Animal (Scientific Procedures) Act 1986. This project licence outlined the program of work and was approved by the University of Edinburgh Animal Welfare Ethical Review Body. Each experimental protocol was approved by the University of Edinburgh veterinary team.

### EAE

WT and KO 9- to 13-week-old female C57BL/6J mice were injected on day 0 subcutaneously in both flanks with 140 μg MOG_35–55_ emulsified in complete Freund’s adjuvant (CFA), and on days 0 and 1 intraperitoneally with 100 μl of 50 ng/ml pertussis toxin in PBS (Hooke Laboratories, #EK-2110). Mice were scored every 2 days until day 6, and then daily based on an EAE scale: 0 –no disease, 1 –flaccid tail, 2 –impaired gait and/or impaired righting reflex, 3 –substantially impaired gait, and 4 –partial hind leg paralysis. Mice with a grade 2 or above were provided with hydrated food on the floor of the cage. Mice with grade 4 were culled immediately.

### Murine tissue and single-cell preparations

Mice were culled via terminal anaesthesia, exsanguination by severing of major vessels and were perfused with PBS. Single-cell preparations of spleens and lymph nodes were achieved by mashing the tissues through a 100-μM strainer and washing with PBS. For the spleen, red blood cells were lysed using RBC Lysis Buffer, as per the manufacturer’s instructions (BD Biosciences, #555899). Preparations of brain and spinal cord were achieved by transferring whole brain and spinal cord to a glass Dounce with 2 ml HBBS and manually homogenising for 50 passes of the Dounce. Cell suspension was centrifuged, and the cell pellet resuspended in 1 ml FBS and 33% Percoll, and 1 ml 10% FBS was layered on top and spun for 15 minutes at 800×*g* at 4°C with no brake. Cells were washed and resuspended in PBS ready for staining.

### Flow cytometry

Cells were stained for surface markers for 30 minutes at 4°C, protected from light. Intracellular cytokines were assessed by incubating cells for 4 hours at 37°C with Cell Stimulation Cocktail containing protein transport inhibitors (eBioscience, #00-4970-03). Cells were fixed, permeabilised, and stained for cytokines using BD Cytofix/Cytoperm (BD Biosciences, #554722) as per the manufacturer’s guidelines. Cells were fixed, permeabilised, and stained for transcription factors using the True-Nuclear Transcription Factor Buffer Set (Biolegend, #424401). Cytokines and transcription factors in the same FACS panel were stained with the FOXP3 transcription factor staining kit (eBioscience, #00-5523-00), as per the manufacturer’s instructions. Cell viability was assessed by flow cytometry fixable live/dead yellow (ThermoFisher #L34959) or Zombie NIR Fixable Viability Kit (Biolegend, #423105).

### Flow cytometry antibodies

#### Mouse

CD45 (clone 30-F11, Biolegend, #103127, 1:200), CD45 (30-F11, BD Biosciences, #564225, 1:200) CD4 (GK1.5, Biolegend, #100406, 1:200), CD4 (GK1.5, Biolegend, #100453, 1:200), CD8 (53–6.7, Biolegend, #100741, 1:200), IL-17A (TC11-18H10.1, Biolegend, #506938, 1:100), IL-17F (9D3.1C8, Biolegend, #517004, 1:100), IFNγ (XMG1.2, Biolegend, #505826, 1:100), CD11c (NF18, Biolegend, #101226, 1:200), CD11b (M1/70, Biolegend, #101212, 1:150), Ly6G (IA8, Biolegend, #127618, 1:200), I-A/I-E (M5/114.15.2, Biolegend, #107635, 1:300), F4/80 (BM8, Biolegend, #123117, 1:200), Ly6C (HK1.4, BD Biosciences, #128021, 1:150), B220 (RA3-6B2, Biolegend, #103245, 1:150), RORγT (B2D, eBioscience, #17-6981-80, 1:100), PD1 (RMP1-30, Biolegend, #109115, 1:300), and CD62L (MEL-14, Biolegend, #104428, 1:300).

#### Human

CD4 (A161A1, Biolegend, #357414, 1:200), CD8 (SK1, Biolegend, #344713, 1:200), and IFNγ (4S.B3, Biolegend, #502517, 1:150).

### NanoString

WT and *Camp*^*−/−*^ KO mice were immunised as previously described. On day 7 post-immunisation, mice were perfused with 10 ml PBS and inguinal lymph nodes were processed as above. DAPI^−^CD3^+^CD4^+^ T cells were sorted using a BD FACSAria II (BD Biosciences), and RNA was extracted immediately using the Qiagen RNAeasy Mini Kit (Qiagen, #74104), as per the manufacturer’s guidelines. Multiplex gene expression analysis (Mouse Immunology Panel) was performed by HTPU Microarray Services, University of Edinburgh. Data analysis was performed using nSolver 4.0 and nCounter Advanced Analysis software.

### Peptides

Synthetic murine cathelicidin (mCRAMP) (GLLRKGGEKIGEKLKKIGQKIKNFFQKLVPQPEQ) and human cathelicidin (LL-37) (LLGDFFRKSKEKIGKEFKRIVQRIKDFLRNLVPRTES) were synthesised by Almac (Penicuik, Scotland) using Fmoc solid phase synthesis and reverse phase HPLC purification. Peptide identity was confirmed by electrospray mass spectrometry. Purity (>95% area) was determined by RP-HPLC and net peptide content determined by amino acid analysis. Lyophilized peptides were reconstituted in endotoxin free water at 5 mg/ml. Reconstituted peptides were tested for endotoxin contamination using a Limulus Amebocyte Lysate Chromogenic Endotoxin Quantitation Kit (Thermo Scientific, UK #88282).

### In vitro plasticity experiments

#### Mouse

Whole splenocytes were prepared by mashing the tissue through a 100-μM strainer and washing with PBS. Red blood cells were lysed using RBC lysis buffer, as per the manufacturer’s instructions (BD Biosciences, #555899). A total of 150,000 cells were plated per well of round-bottom 96-well plates in complete medium (RPMI, 10% foetal calf serum, 10 units/ml penicillin, 10 μg/ml streptomycin, and 2 mM L-glutamine, all supplied by Gibco, Thermo Fisher UK). All cells were differentiated for 48 hours in the presence of plate-bound αCD3 (2.5 μg/ml; Biolegend, #100339), rIL-6 (20 ng/ml; Biolegend, #575706), rIL-23 (20 ng/ml; Biolegend, #589006), and rTGFβ (3 ng/ml; Biolegend, #580706), with or without 2.5 μM mCRAMP. Media was carefully removed and replaced with either rIL-12 (25 ng/ml; Biolegend #575402), with or without 2.5 μM synthetic cathelicidin. Cultures were incubated for a further 72 hours.

In some cultures to assess cathelicidin impact on T cell activation, splenocytes were processed as above and cultured with plate-bound αCD3 (2.5 μg/ml; Biolegend, #100339) in the presence or absence of 2.5 μM mCRAMP for 48 hours.

#### Human

Around 3 ml peripheral venous blood was taken from healthy adult donors in accordance with University of Edinburgh ethical agreements (AMREC 20-HV-069). T cells were isolated immediately using the EasySep direct human T cell isolation kit (StemCell Technologies #19661) according to manufacturer’s instructions. Cells were plated in a 96-well round-bottom plate (2 x 10^5^ per well) in complete medium (RPMI, 10% foetal calf serum, 10 units/ml penicillin, 10 μg/ml streptomycin, and 2 mM L-glutamine, all supplied by Gibco, Thermo Fisher UK). EasySep Immunocult stimulation cocktail (StemCell Technologies #10971) and 3 ng/ml rTGFβ1 (Biolegend #781802) were added to all wells, with or without 2.5 μM synthetic human cathelicidin (LL-37). Five days later, cells were washed twice and replated with 50 ng/ml rIL-12 (Biolegend #573002) again with or without LL-37. Three days later, IFN-γ production was assessed by flow cytometry.

### In vitro restimulation experiments

On day 7 post-immunisation, inguinal lymph nodes were isolated and prepared by mashing the tissue through a 100-μM strainer and washing with PBS. A total of 100,000 cells were plated per well in round-bottom 96-well plates in complete medium (RPMI, 10% foetal calf serum, 10 units/ml penicillin, 10 μg/ml streptomycin, and 2 mM L-glutamine, all supplied by Gibco, Thermo Fisher UK). All cells were incubated for 72 hours with MOG (2.5 μg/ml; Sigma-Aldrich, M4939), with or without rIL-12 (25 ng/ml; Biolegend #575402) and cathelicidin (2.5 μM).

### ELISA

Concentrations of mouse IL-23 (R&D Systems, #DY1887) and IL-12 (R&D Systems #M1270) were determined in spinal cord supernatants 7 and 14 days post-immunisation by ELISA, as per the manufacturer’s guidelines.

### Immunohistochemistry on mouse tissue

Mice were culled by terminal anaesthesia and perfused with 4% paraformaldehyde (PFA) (Sigma-Aldrich, #158127) at indicated time points after immunisation. Whole brain, spinal cord, and inguinal lymph nodes were isolated and fixed in 4% PFA overnight at 4°C. Brains and spinal cords were transferred to 15% sucrose for 24 hours, then 30% sucrose for 24 hours and embedded in optimal cutting temperature (OCT) compound to be cryosectioned (5 μm). Lymph nodes were transferred to 70% ethanol and embedded in paraffin.

Frozen sections were air dried for 10 minutes and paraffin-embedded slides were dewaxed and rehydrated. Haematoxylin–eosin (HE) staining was carried out on tissue sections to understand inflammatory infiltration and the location of lesions within the spinal cord. For spinal cord sections, antigens were retrieved either by microwaving in tri-sodium citrate buffer (1 L distilled H_2_O, 2.94*g* sodium citrate, 0.5 ml Tween20 (pH6)), pH6 for 5 minutes or by 10 minutes incubation with 5 mg/ml proteinase K (Thermo Fisher UK, #AM2548) at 37 degrees. Slides were permeabilised with Triton X-100 (Sigma, #9036-19-5, 1:1,000) and blocked with 25% donkey serum for 1 hour at room temperature (RT), then primary antibodies were incubated in 10% donkey serum overnight at 4°C. Slides were then incubated with a combination of the following secondary antibodies: donkey anti-rat 488 (Invitrogen, #A21208), donkey anti-rabbit 555 (Invitrogen, #A31572), chicken anti-rat 647 (Invitrogen, #A31572), or chicken anti-goat 647 (Invitrogen, #214690) all at 1:500 for 1 hour at RT. Slides were counterstained with Hoechst at 1:1,000 in dH_2_O (Abcam, #H1399). For lymph node sections, antigens were retrieved using 5 mg/ml proteinase K, as above. Slides were blocked with 3% H_2_O_2_, 25% goat serum, and avidin/biotin, and the first primary antibody was added overnight at 4°C. The antigen was visualised using DAB Substrate kit (Vector Labs, #SK-4105) and subsequently blocked with Bloxall Endogenous Blocking Solution (Vector Labs, #SP-6000-100) and 25% horse serum. The second primary antibody was added overnight at 4°C and visualised the following day using Vector Red Substrate Kit (Vector Labs, SK-5100). Slides were counterstained with haematoxylin. Stained sections were mounted in Fluoromount G, scanned on a ZEISS AxioScan.Z1 slide scanner and analysed using ZEISS ZEN software. For NeuN cathelicidin staining, sections were imaged on a Leica SPE confocal microscope.

### Antibodies (mouse)

CRAMP (rabbit polyclonal, Innovagen, #PA-CRPL, 1:500–1:1,000), Ly6G (IA8, Biolegend, #127601, 1:50), CD3 (17A2, Biolegend, #100209, 1:50), CD31 (rat polyclonal, R&D Systems, #AF3628, 1:100), peripheral node addressin (MECA-79, Novus Biologicals, #NB100-77673SS, 1:100), Iba1 (goat polyclonal, Abcam #ab5076, 1:500), F4/80 (BM8, eBioscience, #14–4801 1:100), and NeuN (mouse monoclonal 1B7, Abcam, #ab104224, 1:500).

### Human postmortem brain tissue

Postmortem tissue from secondary progressive MS patients and control patients who died of nonneurological causes, with ethical approval and informed consent, were obtained from the UK Multiple Sclerosis Tissue Bank in collaboration with Professor Anna Williams, University of Edinburgh. Tissue was snap frozen and lesions classified as active, chronic active, chronic inactive, and remyelinating using Luxol Fast Blue-Cresyl Violet staining and Oil Red O staining, according to the International Classification of Neurological Diseases.

### Immunohistochemistry on human tissue

Frozen tissue sections were fixed in 4% PFA and subsequently delipidised in methanol. Antigen retrieval was performed with heating in acid citric buffer and sections blocked with 10% serum. Sections were incubated overnight at 4°C with primary antibodies for CD31 or CD68 (antibodies listed below). On day 2, sections were incubated with the secondary ImmPRESS-AP Horse Anti-Mouse IgG Polymer Detection Kit, Alkaline Phosphatase (Vector, #MP-5402) for 30 minutes at RT and visualised with the Vector Blue Alkaline Phosphatase Substrate Kit (Vector Laboratories #SK-5300). Following this, sections were incubated overnight at 4°C with the second primary antibody for cathelicidin. On day 3, secondary antibody ImmPRESS-AP Horse Anti-Rabbit IgG Polymer Detection Kit Alkaline Phosphatase (Vector, #MP-5401) was added and visualised with the Vector Red Alkaline Phosphatase Substrate Kit (Vector Laboratories #SK-5100). For neutrophil elastase (NE) and cathelicidin staining, frozen tissue sections were fixed in acetone for 5 minutes and delipidised in ethanol for 10 minutes. Antigens were retrieved by heat-induced sodium citrate and permeabilised with Triton X100. True black in 70% ethanol (Cambridge Bioscience #BT2300, dilution 1:20) was added to the slides for 20 seconds and washed thoroughly with PBS and subsequently blocked with 25% donkey serum. Primary antibodies were incubated overnight at 4°C. In all samples, nuclei were counterstained with Hoechst as above and slides mounted with Fluoromount G medium. Relevant IgG isotype antibodies and secondary antibodies alone were used as negative controls.

Cathelicidin-positive cells and NE cathelicidin double-positive cells were manually quantified in MS brains within each lesion type across the whole lesion and calculated as positive cells per cm^2^. The mean area of all lesions was calculated, and 3 white matter areas of this size were counted in control brains.

### Antibodies (human)

Cathelicidin (rabbit polyclonal, abcam #ab69192, dilution 1:100), cathelicidin (rabbit polyclonal, abcam #ab69484, dilution 1:100), NE (clone NP57, Dako M0752, dilution 1:100), CD68 (KP1, Dako M0814, dilution 1:100), and CD31 (JC70A, Dako M0823, dilution 1:100).

### HDP expression in published RNA sequencing databases

In mice and humans, published sequencing databases were identified using searches in Google Scholar, PubMed, and GEO Accession Viewer. The search terms for the datasets were as follows: “cell type” AND “rna seq” AND “human/mouse” NOT “iPSC.” The search was filtered to include series entry type only. For data deposited on a laboratory website, the HDP gene name was entered into the search engine and gene expression assessed. *cd3g/*CD3G was used as a negative control reference gene that encodes for CD3, which is a protein complex expressed only on T cells, therefore is not expressed by CNS-resident cells.

### Statistics

Two groups were compared with two-way unpaired or paired *t* test. Multiple groups were compared by one- or two-way ANOVA analysis. All data were analysed with Graphpad Prism. Sample sizes and what each data point on the graphs represent are detailed in the figure legends.

## Supporting information

S1 Fig Establishment of the EAE model in WT mice. WT mice were immunised with MOG in CFA then pertussis toxin as previously (Fig 1). Mice were (A) tracked for clinical signs of disease over a time course and (B) at various times were perfused with 4% PFA and spinal cords removed. HE staining was performed to detect inflammatory infiltrate and (C, D) anti-CD3 immunofluorescent staining performed to detect T cell infiltrate. (E) Representative plot of IL-17A^+^ CD4^+^ T cells in the spinal cord on day 14. The graph shows Th17 cell numbers (IL-17A^+^ and IL-17F^+^) in the spinal cord quantified by flow cytometry. Data shown are (A) mean with standard error and (D, E) individual data points with line at median. N values: A– 85 mice; D– 14–50 sections from 3–4 mice; E– 4. Images are representative of 3 mice. Statistical test used in D–one-way ANOVA. Data available at 10.6084/m9.figshare.20310363. CFA, complete Freund’s adjuvant; EAE, experimental autoimmune encephalomyelitis; HE, haematoxylin–eosin; MOG, myelin oligodendrocyte glycoprotein; PFA, paraformaldehyde; WT, wild-type.
(DOCX)

S2 Fig Generation and validation of the LysMCre mouse. The WT *Camp* gene (A) consists of 4 exons on chromosome 9. *Camp*^*tm1a(EUCOMM)Hmgo*^ ES cells (JM8A3.N1; cell clone ID HEPD0722_1_E10; MGI:4950203) cells targeting the *Camp* locus were purchased from EUCOMM, injected into C57/Bl6J blastocyst stage embryos and subsequently transferred to recipient female mice. Male chimeric progeny were mated with C57Bl/6JCrl female mice to establish germ line transmission, confirmed by short range and long range PCR (Primer combinations and sequences in Methods). Targeted mice (*Camp*^*tm1a(EUCOMM)Hmgo*^; B) were then crossed with ActFlpE (SJL-Tg(ACTFLPe)9205Dym/J) mice on C57Bl6/JCrl background mice to generate mice with a “flipped” allele (*Camp*^*tm1a(EUCOMM)Hmgo / ACTFLPe*^; C) lacking the lacZ and neo vector cassettes, intercrossed to homozygosity and confirmed by PCR. *C*onfirmation of Cre recombination to generate a conditional null allele (with excision of *Camp* exons 2–4) was provided by administration of soluble tat Cre recombinase to one cell *Camp*^*tm1a(EUCOMM)Hmgo / ACTFLPe*^ embryos obtained by IVF, transferred into recipients at 2.5 days, followed by PCR confirmation and sequencing of the resulting conditional null allele on E12 embryos. *Camp*^*tm1a(EUCOMM)Hmgo / ACTFLPe*^ were crossed with a myeloid-specific CRE-recombinase line LysMCre(Tg (Lyz2tm1(cre)Ifo) to generate the *Camp* conditional null mice (*Camp*^*tm1a(EUCOMM)Hmgo / ACTFLPe / Tg (Lyz2tm1(cre)Ifo*^; D) designated *LysMCreFLCamp* mice and bred to congenicity for *n =* 10 generations. Breeding pairs homozygous for floxed *Camp* were heterozygous or WT for LysMCre (designated *LysMCreFLCamp* and *FLCamp*, respectively) and studied experimentally compared to littermates controls. (E) Primers and (F) sequences used.
(DOCX)

S3 Fig **Gating strategy for (A) CD4+ T cells and (B) other leukocyte populations in the spinal cord**.
(DOCX)

S4 Fig Analysis of T cell populations in untreated WT C57BL/6J and Camp KO mice. Mice were culled between 6 and 12 weeks of age and inguinal lymph nodes (B, C, and E) and spleens (B, C, and F) were harvested and analysed by flow cytometry immediately. CD4^+^ and CD8^+^ T cell frequency and expression of activation markers PD-1 and CD62L in these organs were assessed in both genotypes. Representative plots of CD4^+^ and CD8^+^ T cell frequency are shown and the graph shows T cell frequency quantified by flow cytometry (A-C). Representative plots of PD1 expression on CD4^+^ T cells are shown (A) and the graph shows activation marker expression on CD4^+^ T cells in the inguinal lymph node (E) and spleen (F). N values: 3–8 mice per genotype. KO, knockout; WT, wild-type.
(DOCX)

S1 Table Murine neuron and astrocyte databases accessed for analysis of HDP expression in CNS-resident cells. The cell type, data availability information, reference, CNS region, and experimental condition is listed. Data available at 10.6084/m9.figshare.20310363. CNS, central nervous system; HDP, host-defence peptide.
(DOCX)

S2 Table Murine microglia and oligodendrocyte databases accessed for analysis of HDP expression in CNS-resident cells. The cell type, data availability information, reference, CNS region and experimental condition is listed. CNS, central nervous system; HDP, host-defence peptide.
(DOCX)

S3 Table Information relating to human tissue donors. MS, multiple sclerosis; PP, primary progressive; SP, secondary progressive.
(DOCX)

S4 Table Human neuron, astrocyte, microglia, and oligodendrocyte databases accessed for analysis of HDP expression in CNS-resident cells. The cell type, data availability information, reference, CNS region, and experimental condition is listed. CNS, central nervous system; HDP, host-defence peptide.
(DOCX)

## Abbreviations

BBB: blood–brain barrier
CFA: complete Freund’s adjuvant
CNS: central nervous system
EAE: experimental autoimmune encephalomyelitis
HE: haematoxylin–eosin
KO: knockout
MOG: myelin oligodendrocyte glycoprotein
MS: multiple sclerosis
NE: neutrophil elastase
PFA: paraformaldehyde
RT: room temperature
WT: wild-type
