# Supplementary material for: The antimicrobial peptide cathelicidin drives development of experimental autoimmune encephalomyelitis in mice by affecting Th17 differentiation
Source: PLoS Biol. 2022 Aug 26;20(8):e3001554. doi: 10.1371/journal.pbio.3001554 (PMC9455863; doi:10.1371/journal.pbio.3001554)
Supplement: S1 Fig — WT mice were immunised with MOG in CFA then pertussis toxin as previously (Fig 1). Mice were (A) tracked for clinical signs of disease over a time course and (B) at various times were perfused with 4% PFA and spinal cords removed. HE staining was performed to detect inflammatory infiltrate and (C, D) anti-CD3 immunofluorescent staining performed to detect T cell infiltrate. (E) Representative plot of IL-17A+ CD4+ T cells in the spinal cord on day 14. The graph shows Th17 cell numbers (IL-17A+ and IL-17F+) in the spinal cord quantified by flow cytometry. Data shown are (A) mean with standard error and (D, E) individual data points with line at median. N values: A– 85 mice; D– 14–50 sections from 3–4 mice; E– 4. Images are representative of 3 mice. Statistical test used in D–one-way ANOVA. Data available at 10.6084/m9.figshare.20310363. CFA, complete Freund’s adjuvant; EAE, experimental autoimmune encephalomyelitis; HE, haematoxylin–eosin; MOG, myelin oligodendrocyte glycoprotein; PFA, paraformaldehyde; WT, wild-type. (DOCX) [file pbio.3001554.s001.docx]

**Supporting Information S1_Fig**

**The antimicrobial peptide cathelicidin is critical for the development of Th17 responses in experimental autoimmune encephalomyelitis**


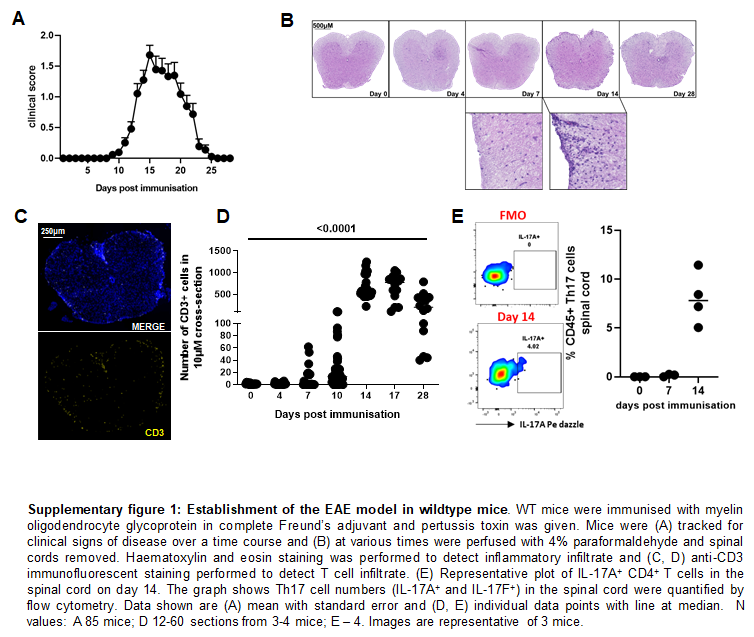
Katie J Smith^1^, Danielle Minns^1^, Brian J McHugh^1^, Rebecca K. Holloway^2,3^, Richard O’Connor^1^, Anna Williams^3^, Lauren Melrose^1^, Rhoanne McPherson^1^, Veronique E. Miron^2^, Donald J Davidson^1^and Emily Gwyer Findlay^1^
