# Supplementary material for: The antimicrobial peptide cathelicidin drives development of experimental autoimmune encephalomyelitis in mice by affecting Th17 differentiation
Source: PLoS Biol. 2022 Aug 26;20(8):e3001554. doi: 10.1371/journal.pbio.3001554 (PMC9455863; doi:10.1371/journal.pbio.3001554)
Supplement: S2 Fig — The WT Camp gene (A) consists of 4 exons on chromosome 9. Camptm1a(EUCOMM)Hmgo ES cells (JM8A3.N1; cell clone ID HEPD0722_1_E10; MGI:4950203) cells targeting the Camp locus were purchased from EUCOMM, injected into C57/Bl6J blastocyst stage embryos and subsequently transferred to recipient female mice. Male chimeric progeny were mated with C57Bl/6JCrl female mice to establish germ line transmission, confirmed by short range and long range PCR (Primer combinations and sequences in Methods). Targeted mice (Camptm1a(EUCOMM)Hmgo; B) were then crossed with ActFlpE (SJL-Tg(ACTFLPe)9205Dym/J) mice on C57Bl6/JCrl background mice to generate mice with a “flipped” allele (Camptm1a(EUCOMM)Hmgo / ACTFLPe; C) lacking the lacZ and neo vector cassettes, intercrossed to homozygosity and confirmed by PCR. Confirmation of Cre recombination to generate a conditional null allele (with excision of Camp exons 2–4) was provided by administration of soluble tat Cre recombinase to one cell Camptm1a(EUCOMM)Hmgo / ACTFLPe embryos obtained by IVF, transferred into recipients at 2.5 days, followed by PCR confirmation and sequencing of the resulting conditional null allele on E12 embryos. Camptm1a(EUCOMM)Hmgo / ACTFLPe were crossed with a myeloid-specific CRE-recombinase line LysMCre(Tg (Lyz2tm1(cre)Ifo) to generate the Camp conditional null mice (Camptm1a(EUCOMM)Hmgo / ACTFLPe / Tg (Lyz2tm1(cre)Ifo; D) designated LysMCreFLCamp mice and bred to congenicity for n = 10 generations. Breeding pairs homozygous for floxed Camp were heterozygous or WT for LysMCre (designated LysMCreFLCamp and FLCamp, respectively) and studied experimentally compared to littermates controls. (E) Primers and (F) sequences used. (DOCX) [file pbio.3001554.s002.docx]

**Supporting Information S2_Fig**

**The antimicrobial peptide cathelicidin is critical for the development of Th17 responses in experimental autoimmune encephalomyelitis**


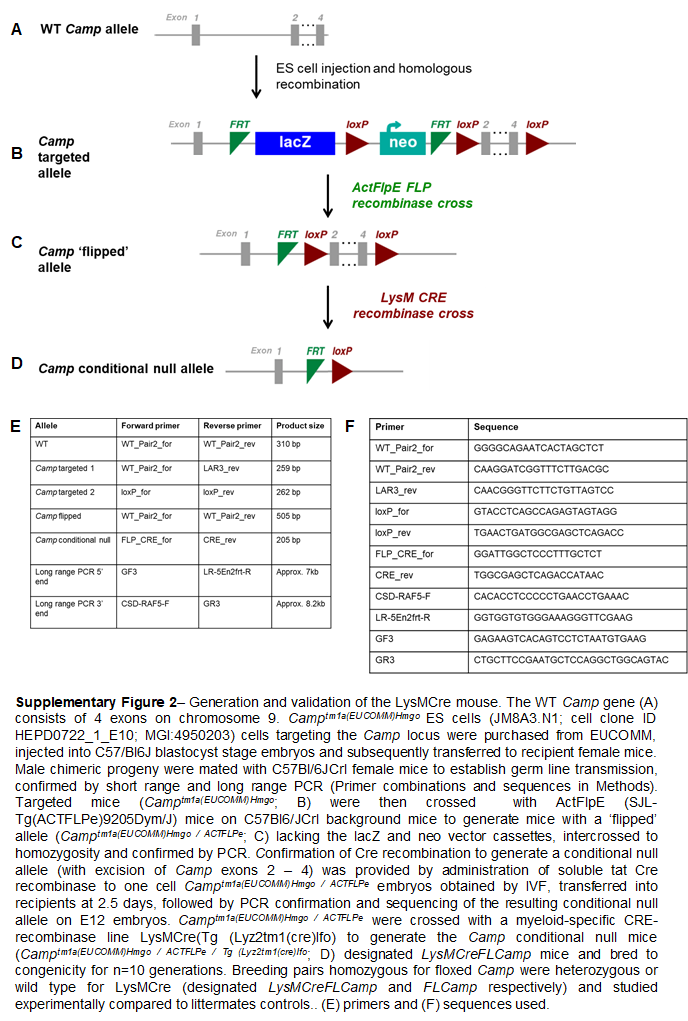
Katie J Smith^1^, Danielle Minns^1^, Brian J McHugh^1^, Rebecca K. Holloway^2,3^, Richard O’Connor^1^, Anna Williams^3^, Lauren Melrose^1^, Rhoanne McPherson^1^, Veronique E. Miron^2^, Donald J Davidson^1^and Emily Gwyer Findlay^1^
