# Supplementary material for: The antimicrobial peptide cathelicidin drives development of experimental autoimmune encephalomyelitis in mice by affecting Th17 differentiation
Source: PLoS Biol. 2022 Aug 26;20(8):e3001554. doi: 10.1371/journal.pbio.3001554 (PMC9455863; doi:10.1371/journal.pbio.3001554)

**Supporting Information S3_Fig**

**The antimicrobial peptide cathelicidin is critical for the development of Th17 responses in experimental autoimmune encephalomyelitis**

Katie J Smith^1^, Danielle Minns^1^, Brian J McHugh^1^, Rebecca K. Holloway^2,3^, Richard O’Connor^1^, Anna Williams^3^, Lauren Melrose^1^, Rhoanne McPherson^1^, Veronique E. Miron^2^, Donald J Davidson^1^and Emily Gwyer Findlay^1^


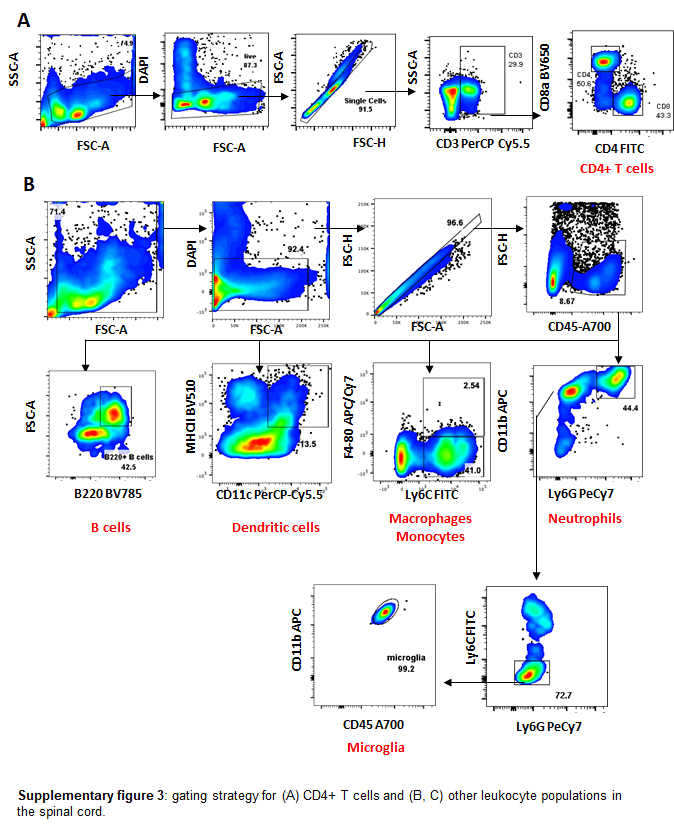

Supplement: S3 Fig — Gating strategy for (A) CD4+ T cells and (B) other leukocyte populations in the spinal cord. (DOCX) [file pbio.3001554.s003.docx]
