# Supplementary material for: The antimicrobial peptide cathelicidin drives development of experimental autoimmune encephalomyelitis in mice by affecting Th17 differentiation
Source: PLoS Biol. 2022 Aug 26;20(8):e3001554. doi: 10.1371/journal.pbio.3001554 (PMC9455863; doi:10.1371/journal.pbio.3001554)
Supplement: S4 Fig — Mice were culled between 6 and 12 weeks of age and inguinal lymph nodes (B, C, and E) and spleens (B, C, and F) were harvested and analysed by flow cytometry immediately. CD4+ and CD8+ T cell frequency and expression of activation markers PD-1 and CD62L in these organs were assessed in both genotypes. Representative plots of CD4+ and CD8+ T cell frequency are shown and the graph shows T cell frequency quantified by flow cytometry (A-C). Representative plots of PD1 expression on CD4+ T cells are shown (A) and the graph shows activation marker expression on CD4+ T cells in the inguinal lymph node (E) and spleen (F). N values: 3–8 mice per genotype. KO, knockout; WT, wild-type. (DOCX) [file pbio.3001554.s004.docx]

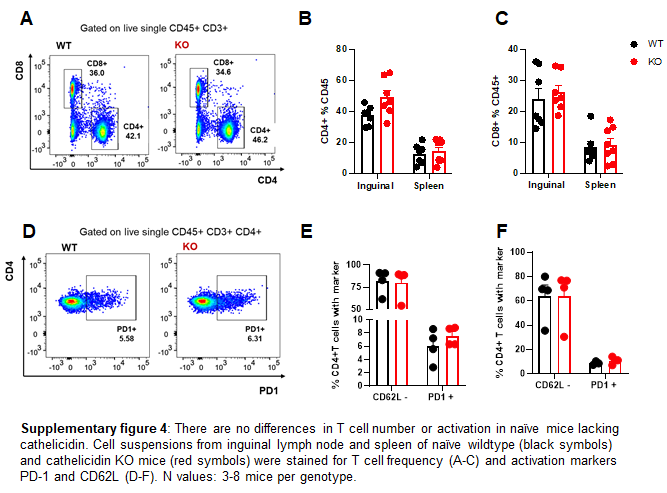


**Supporting Information S4_Fig**

**The antimicrobial peptide cathelicidin is critical for the development of Th17 responses in experimental autoimmune encephalomyelitis**

Katie J Smith^1^, Danielle Minns^1^, Brian J McHugh^1^, Rebecca K. Holloway^2,3^, Richard O’Connor^1^, Anna Williams^3^, Lauren Melrose^1^, Rhoanne McPherson^1^, Veronique E. Miron^2^, Donald J Davidson^1^and Emily Gwyer Findlay^1^
