# Supplementary material for: The antimicrobial peptide cathelicidin drives development of experimental autoimmune encephalomyelitis in mice by affecting Th17 differentiation
Source: PLoS Biol. 2022 Aug 26;20(8):e3001554. doi: 10.1371/journal.pbio.3001554 (PMC9455863; doi:10.1371/journal.pbio.3001554)
Supplement: S1 Table — The cell type, data availability information, reference, CNS region, and experimental condition is listed. Data available at 10.6084/m9.figshare.20310363. CNS, central nervous system; HDP, host-defence peptide. (DOCX) [file pbio.3001554.s005.docx]

**Supporting Information S1_Table**

**The antimicrobial peptide cathelicidin is critical for the development of Th17 responses in experimental autoimmune encephalomyelitis**

Katie J Smith^1^, Danielle Minns^1^, Brian J McHugh^1^, Rebecca K. Holloway^2,3^, Richard O’Connor^1^, Anna Williams^3^, Lauren Melrose^1^, Rhoanne McPherson^1^, Veronique E. Miron^2^, Donald J Davidson^1^and Emily Gwyer Findlay^1^

| Cell type | Data availability | Reference | Region | Condition |
| --- | --- | --- | --- | --- |
| Neurons | GSE100175 | (1) | Trigemenal neurons compared to DRG neurons | Following cell sort |
|  | GSE62424 | (2) | Intact DRG, acutely dissociated DRG and magnetically-purified DRG neurons | Following cell sort |
|  | GSE63576 | (3) | Somatosensory Neurons | Steady state |
|  | GSE77067 | (4) | Dentate granule cells | Saline-treated mouse in homecage or novel environment |
|  | GSE71485 | (5) | Neurons in L4 of adult mouse visual cortex | Steady state |
|  | GSE52564 | (6) | Cerebral cortex | Purified populations of neurons from cerebral cortex during steady-state |
|  | N/A | (7) | Dorsal root ganglia (whole sensory ganglia) | Database allowing for comparison of gene expression to other tissue types  https://bbs.utdallas.edu/painneurosciencelab/sensoryomics/drgtxome/ |
|  | GSE115997 | (8) | Light sensitive neurons in the primary visual cortex (V1) | Light stimulation or non-light stimulation |
|  | GSE140946 | (9) | Clonal excitatory neurons | Patch-sequencing on neurons from Nestin-positive progenitors in the neocortex |
|  | GSE134378 | (10) | Neurons in L4 of adult mouse visual cortex | Steady state |
|  | GSE130950 | (11) | Parvalbumin positive interneurons | Steady state |
|  | GSE70844 | (12) | Cortical neurons | Following whole-cell patch-clamp recordings |
|  | GSE146043 | (13) | Olfactory sensory neurons | Steady-state |
|  | GSE136396 | (14) | Cold-sensitive or cold-insensitive neurons | Steady-state |
|  | GSE120284 | (15) | DRG | FACS-sorted glial cells following nerve ligation |
|  | GSE102962 | (16) | Colonic sensory neurons Peripheral nervous system | Steady-state |
|  | GSE52564 | (17) | Juvenile and adult human brain | Steady-state |
|  | GSE149154 | (18) | Human Aggregcan-positive interneurons Puramidal neurons | Post-mortem brain tissue |
|  |  | (7) | Human DRG | DRG |
| Astrocytes | GSE76097 | (19) | Spinal cord astrocytes | Laminectomy of single vertebra |
|  |  | (20) | Cortex Hippocampus Striatum | Different brain regions across the life-san of the mouse |
|  | GSE99791 | (21) | Visual cortex, somatomotor cortex, hypothalamus, and cerebellum. | Multiple brain regions from 4-month and 2-years old mice |
|  | GSE94010 | (22) | Cortex Hippocampus Striatum | Comparing adult striatal and hippocampal astrocytes under identical conditions  http://astrocyternaseq.org |
|  | GSE52564 | (6) | Cerebral cortex | Purified populations of neurons from cerebral cortex during steady-state |
|  | GSE114000 | (23) | Cortex Hippocampus | Assessing molecular diversity of astrocytes across forebrain regions |
|  | Supplementary data in paper | (24) | Brain stem, cortex and olfactory bulb | Investigating region-specific gene signatures of astrocytes in steady state |
|  | GSE143282 | (25) | Olfactory bulb, hippocampus, and brainstem | Aldh1l1-GFP positive astrocytes in the cortex of 16-week old mice |

1. Lopes DM, Denk F, McMahon SB. The Molecular Fingerprint of Dorsal Root and Trigeminal Ganglion Neurons. Front Mol Neurosci. 2017;10:304.

2. Thakur M, Crow M, Richards N, Davey GI, Levine E, Kelleher JH, et al. Defining the nociceptor transcriptome. Front Mol Neurosci. 2014;7:87.

3. Li CL, Li KC, Wu D, Chen Y, Luo H, Zhao JR, et al. Somatosensory neuron types identified by high-coverage single-cell RNA-sequencing and functional heterogeneity. Cell Res. 2016;26(1):83-102.

4. Lacar B, Linker SB, Jaeger BN, Krishnaswami SR, Barron JJ, Kelder MJE, et al. Nuclear RNA-seq of single neurons reveals molecular signatures of activation. Nat Commun. 2016;7:11022.

5. Shin J, Berg DA, Zhu Y, Shin JY, Song J, Bonaguidi MA, et al. Single-Cell RNA-Seq with Waterfall Reveals Molecular Cascades underlying Adult Neurogenesis. Cell Stem Cell. 2015;17(3):360-72.

6. Zhang Y, Chen K, Sloan SA, Bennett ML, Scholze AR, O'Keeffe S, et al. An RNA-sequencing transcriptome and splicing database of glia, neurons, and vascular cells of the cerebral cortex. J Neurosci. 2014;34(36):11929-47.

7. Ray P, Torck A, Quigley L, Wangzhou A, Neiman M, Rao C, et al. Comparative transcriptome profiling of the human and mouse dorsal root ganglia: an RNA-seq–based resource for pain and sensory neuroscience research. PAIN. 2018;159(7).

8. Liu J, Wang M, Sun L, Pan NC, Zhang C, Zhang J, et al. Integrative analysis of in vivo recording with single-cell RNA-seq data reveals molecular properties of light-sensitive neurons in mouse V1. Protein Cell. 2020;11(6):417-32.

9. Cadwell CR, Scala F, Fahey PG, Kobak D, Mulherkar S, Sinz FH, et al. Cell type composition and circuit organization of clonally related excitatory neurons in the juvenile mouse neocortex. Elife. 2020;9.

10. Scala F, Kobak D, Shan S, Bernaerts Y, Laturnus S, Cadwell CR, et al. Layer 4 of mouse neocortex differs in cell types and circuit organization between sensory areas. Nat Commun. 2019;10(1):4174.

11. Bomkamp C, Tripathy SJ, Bengtsson Gonzales C, Hjerling-Leffler J, Craig AM, Pavlidis P. Transcriptomic correlates of electrophysiological and morphological diversity within and across excitatory and inhibitory neuron classes. PLoS Comput Biol. 2019;15(6):e1007113.

12. Fuzik J, Zeisel A, Máté Z, Calvigioni D, Yanagawa Y, Szabó G, et al. Integration of electrophysiological recordings with single-cell RNA-seq data identifies neuronal subtypes. Nat Biotechnol. 2016;34(2):175-83.

13. Tan K, Jones SH, Lake BB, Dumdie JN, Shum EY, Zhang L, et al. The role of the NMD factor UPF3B in olfactory sensory neurons. Elife. 2020;9.

14. Feketa VV, Nikolaev YA, Merriman DK, Bagriantsev SN, Gracheva EO. CNGA3 acts as a cold sensor in hypothalamic neurons. Elife. 2020;9.

15. Jager SE, Pallesen LT, Richner M, Harley P, Hore Z, McMahon S, et al. Changes in the transcriptional fingerprint of satellite glial cells following peripheral nerve injury. Glia. 2020;68(7):1375-95.

16. Hockley JRF, Taylor TS, Callejo G, Wilbrey AL, Gutteridge A, Bach K, et al. Single-cell RNAseq reveals seven classes of colonic sensory neuron. Gut. 2019;68(4):633-44.

17. Zhang Y, Sloan SA, Clarke LE, Caneda C, Plaza CA, Blumenthal PD, et al. Purification and Characterization of Progenitor and Mature Human Astrocytes Reveals Transcriptional and Functional Differences with Mouse. Neuron. 2016;89(1):37-53.

18. Garst-Orozco J, Malik R, Lanz TA, Weber ML, Xi H, Arion D, et al. GluN2D-mediated excitatory drive onto medial prefrontal cortical PV+ fast-spiking inhibitory interneurons. PLoS One. 2020;15(6):e0233895.

19. Anderson MA, Burda JE, Ren Y, Ao Y, O'Shea TM, Kawaguchi R, et al. Astrocyte scar formation aids central nervous system axon regeneration. Nature. 2016;532(7598):195-200.

20. Clarke LE, Liddelow SA, Chakraborty C, Münch AE, Heiman M, Barres BA. Normal aging induces A1-like astrocyte reactivity. Proc Natl Acad Sci U S A. 2018;115(8):E1896-e905.

21. Boisvert MM, Erikson GA, Shokhirev MN, Allen NJ. The Aging Astrocyte Transcriptome from Multiple Regions of the Mouse Brain. Cell Rep. 2018;22(1):269-85.

22. Chai H, Diaz-Castro B, Shigetomi E, Monte E, Octeau JC, Yu X, et al. Neural Circuit-Specialized Astrocytes: Transcriptomic, Proteomic, Morphological, and Functional Evidence. Neuron. 2017;95(3):531-49.e9.

23. Batiuk MY, Martirosyan A, Wahis J, de Vin F, Marneffe C, Kusserow C, et al. Identification of region-specific astrocyte subtypes at single cell resolution. Nat Commun. 2020;11(1):1220.

24. Cuevas-Diaz Duran R, Wang C-Y, Zheng H, Deneen B, Wu JQ. Brain Region-Specific Gene Signatures Revealed by Distinct Astrocyte Subpopulations Unveil Links to Glioma and Neurodegenerative Diseases. eneuro. 2019;6(2):ENEURO.0288-18.2019.

25. Lozzi B, Huang TW, Sardar D, Huang AY, Deneen B. Regionally Distinct Astrocytes Display Unique Transcription Factor Profiles in the Adult Brain. Front Neurosci. 2020;14:61.
