# Supplementary material for: The antimicrobial peptide cathelicidin drives development of experimental autoimmune encephalomyelitis in mice by affecting Th17 differentiation
Source: PLoS Biol. 2022 Aug 26;20(8):e3001554. doi: 10.1371/journal.pbio.3001554 (PMC9455863; doi:10.1371/journal.pbio.3001554)
Supplement: S2 Table — The cell type, data availability information, reference, CNS region and experimental condition is listed. CNS, central nervous system; HDP, host-defence peptide. (DOCX) [file pbio.3001554.s006.docx]

**Supporting Information S2_Table**

**The antimicrobial peptide cathelicidin is critical for the development of Th17 responses in experimental autoimmune encephalomyelitis**

Katie J Smith^1^, Danielle Minns^1^, Brian J McHugh^1^, Rebecca K. Holloway^2,3^, Richard O’Connor^1^, Anna Williams^3^, Lauren Melrose^1^, Rhoanne McPherson^1^, Veronique E. Miron^2^, Donald J Davidson^1^and Emily Gwyer Findlay^1^

| Cell type | Data availability | Reference | Region | Condition |
| --- | --- | --- | --- | --- |
| Microglia | <http://www.brainrnaseq.org> | (1) | Whole brain or regions | Purified populations of microglia from cerebral cortex during steady-state |
|  |  | (2) | Whole brain, meninges, dura mater and choroid plexus | Isolated brains or border regions from WT homeostatic mouse brain |
|  | GSE71133 | (3) | Spinal cord | Spinal cord microglia following spinal nerve ligation |
|  | GSE52564 | (4) | Cerebral cortex | RNA sequencing of mouse microglia across various timepoints |
|  | GSE75431 | (5) | Whole brain | Microglia from 7- or 13-month old PS2APP or non-transgenic mice |
|  | GSE65067 | (6) | Hippocampi and cortices | WT or TREM2-deficient microglia in 5xFAD model |
|  | GSE74615 | (7) | Cortex | Microglia in APPswe/PS1dE9 model and aged controls |
|  | GSE77986 | (8) | Two ipsilesional hemispheres with cerebellum and brainstem removed | Wildtype and Clec4e-/- mice after 1h of transient middle cerebral artery occlusion and 25h of reperfusion |
|  | GSE66926 | (9) | Whole brain | Wildtype and TREM2-deficient microglia in response to cuprizone-mediated demyelinatin |
|  | GSE67858 | (10) | Whole brain | FACS-sorted microglia from LPS-injected or LCMV-infected mice |
|  | GSE68376 | (11) | Whole brain | Sequenced wildtype microglia, repopulating microglia following ablation and bone marrow-derived macrophages |
|  | GSE62420 | (12) | Cerebellul, cortex, hippocampus and striatum | Steady-state 4-, 12-, and 22-month old mice |
|  | GSE79812 | (13) | Brain without brainstem and meninges | Yolk sac progenitors, microglia from early brain and microglia from later stages were isolated from CX3CR1+ C58BL/6J mice or microglia from perturbation models |
| Oligodendrocytes | GSE75330 | (14) | Anterior-posterior and dorsal-ventral axis | RNA sequencing in the mouse juvenile CNS http://linnarssonlab.org/oligodendrocytes/ |
|  | GSE95194 | (15) | Forebrain and spinal cord | Timepoints E13.5, P7, juvenile and adult mice https://castelobranco.shinyapps.io/OPCsinglecell2017/ |
|  | GSE52564 | (4) | Cerebral cortex | RNA sequencing of mouse oligodendrocytes across various timepoints |
|  | GSE128525 | (16) | Spinal cord | OL lineage from Sox10::Cre-GFP mice following spinal cord injury  https://castelobranco.shinyapps.io/ltCC_iSC2/ |
|  | GSE101915 | (17) | Cerebellum | RNA sequencing on cells of the mouse cerebellum in steady state |
|  | GSE145044 | Not yet published | Corpus callosum | Following LAQ treatment, with controls receiving no treatment |
|  | GSE119127 | (18) | Forebrain | Ep400-deficient mouse oligodendrocytes following culture |
|  | GSE94067 | (19) |  | OPCs with or without PRMT5 expression |

1. Li Q, Cheng Z, Zhou L, Darmanis S, Neff NF, Okamoto J, et al. Developmental Heterogeneity of Microglia and Brain Myeloid Cells Revealed by Deep Single-Cell RNA Sequencing. Neuron. 2019;101(2):207-23.e10.

2. Van Hove H, Martens L, Scheyltjens I, De Vlaminck K, Pombo Antunes AR, De Prijck S, et al. A single-cell atlas of mouse brain macrophages reveals unique transcriptional identities shaped by ontogeny and tissue environment. Nature Neuroscience. 2019;22(6):1021-35.

3. Denk F, Crow M, Didangelos A, Lopes DM, McMahon SB. Persistent Alterations in Microglial Enhancers in a Model of Chronic Pain. Cell Rep. 2016;15(8):1771-81.

4. Zhang Y, Chen K, Sloan SA, Bennett ML, Scholze AR, O'Keeffe S, et al. An RNA-sequencing transcriptome and splicing database of glia, neurons, and vascular cells of the cerebral cortex. J Neurosci. 2014;34(36):11929-47.

5. Srinivasan K, Friedman BA, Larson JL, Lauffer BE, Goldstein LD, Appling LL, et al. Untangling the brain's neuroinflammatory and neurodegenerative transcriptional responses. Nat Commun. 2016;7:11295.

6. Wang Y, Cella M, Mallinson K, Ulrich JD, Young KL, Robinette ML, et al. TREM2 lipid sensing sustains the microglial response in an Alzheimer's disease model. Cell. 2015;160(6):1061-71.

7. Orre M, Kamphuis W, Osborn LM, Jansen AHP, Kooijman L, Bossers K, et al. Isolation of glia from Alzheimer's mice reveals inflammation and dysfunction. Neurobiol Aging. 2014;35(12):2746-60.

8. Arumugam TV, Manzanero S, Furtado M, Biggins PJ, Hsieh YH, Gelderblom M, et al. An atypical role for the myeloid receptor Mincle in central nervous system injury. J Cereb Blood Flow Metab. 2017;37(6):2098-111.

9. Poliani PL, Wang Y, Fontana E, Robinette ML, Yamanishi Y, Gilfillan S, et al. TREM2 sustains microglial expansion during aging and response to demyelination. J Clin Invest. 2015;125(5):2161-70.

10. Erny D, Hrabě de Angelis AL, Jaitin D, Wieghofer P, Staszewski O, David E, et al. Host microbiota constantly control maturation and function of microglia in the CNS. Nat Neurosci. 2015;18(7):965-77.

11. Bruttger J, Karram K, Wörtge S, Regen T, Marini F, Hoppmann N, et al. Genetic Cell Ablation Reveals Clusters of Local Self-Renewing Microglia in the Mammalian Central Nervous System. Immunity. 2015;43(1):92-106.

12. Grabert K, Michoel T, Karavolos MH, Clohisey S, Baillie JK, Stevens MP, et al. Microglial brain region-dependent diversity and selective regional sensitivities to aging. Nat Neurosci. 2016;19(3):504-16.

13. Matcovitch-Natan O, Winter DR, Giladi A, Vargas Aguilar S, Spinrad A, Sarrazin S, et al. Microglia development follows a stepwise program to regulate brain homeostasis. Science. 2016;353(6301):aad8670.

14. Marques S, Zeisel A, Codeluppi S, van Bruggen D, Mendanha Falcão A, Xiao L, et al. Oligodendrocyte heterogeneity in the mouse juvenile and adult central nervous system. Science. 2016;352(6291):1326-9.

15. Marques S, van Bruggen D, Vanichkina DP, Floriddia EM, Munguba H, Väremo L, et al. Transcriptional Convergence of Oligodendrocyte Lineage Progenitors during Development. Dev Cell. 2018;46(4):504-17.e7.

16. Floriddia EM, Lourenço T, Zhang S, van Bruggen D, Hilscher MM, Kukanja P, et al. Distinct oligodendrocyte populations have spatial preference and different responses to spinal cord injury. Nat Commun. 2020;11(1):5860.

17. Xu X, Stoyanova EI, Lemiesz AE, Xing J, Mash DC, Heintz N. Species and cell-type properties of classically defined human and rodent neurons and glia. Elife. 2018;7.

18. Elsesser O, Fröb F, Küspert M, Tamm ER, Fujii T, Fukunaga R, et al. Chromatin remodeler Ep400 ensures oligodendrocyte survival and is required for myelination in the vertebrate central nervous system. Nucleic Acids Res. 2019;47(12):6208-24.

19. Scaglione A, Patzig J, Liang J, Frawley R, Bok J, Mela A, et al. PRMT5-mediated regulation of developmental myelination. Nat Commun. 2018;9(1):2840.
