# Supplementary material for: The antimicrobial peptide cathelicidin drives development of experimental autoimmune encephalomyelitis in mice by affecting Th17 differentiation
Source: PLoS Biol. 2022 Aug 26;20(8):e3001554. doi: 10.1371/journal.pbio.3001554 (PMC9455863; doi:10.1371/journal.pbio.3001554)
Supplement: S3 Table — MS, multiple sclerosis; PP, primary progressive; SP, secondary progressive. (DOCX) [file pbio.3001554.s007.docx]

| **MS Patients** | **Sex** | **Age (Years)** | **MS Classification** | **Disease Duration (Years)** | **Post-mortem Interval (Hours)** |
| --- | --- | --- | --- | --- | --- |
| MS100 | M | 46 | SP | 8 | 7 |
| MS136 | M | 40 | SP | 9 | 10 |
| MS154 | F | 34 | SP | 21 | 12 |
| MS176 | M | 37 | PP | 27 | 12 |
| MS207 | F | 46 | SP | 25 | 10 |
| MS230 | F | 42 | SP | 19 | 31 |
| MS122 | M | 44 | SP | Not known | 16 |
|  |  |  |  |  |  |
| **Controls** | **Sex** | **Age (Years)** | **Cause of Death** | **Post-mortem Interval** |  |
| CO25 | M | 35 | Carcinoma of the tongue | 22 |  |
| CO28 | F | 60 | Ovarian Cancer | 13 |  |
| CO39 | M | 82 | Myelodysplastic syndrome, Rheumatoid arthritis | 21 |  |

**Supporting Information S3_Table**

**The antimicrobial peptide cathelicidin is critical for the development of Th17 responses in experimental autoimmune encephalomyelitis**

Katie J Smith^1^, Danielle Minns^1^, Brian J McHugh^1^, Rebecca K. Holloway^2,3^, Richard O’Connor^1^, Anna Williams^3^, Lauren Melrose^1^, Rhoanne McPherson^1^, Veronique E. Miron^2^, Donald J Davidson^1^and Emily Gwyer Findlay^1^
