# Supplementary material for: The antimicrobial peptide cathelicidin drives development of experimental autoimmune encephalomyelitis in mice by affecting Th17 differentiation
Source: PLoS Biol. 2022 Aug 26;20(8):e3001554. doi: 10.1371/journal.pbio.3001554 (PMC9455863; doi:10.1371/journal.pbio.3001554)
Supplement: S4 Table — The cell type, data availability information, reference, CNS region, and experimental condition is listed. CNS, central nervous system; HDP, host-defence peptide. (DOCX) [file pbio.3001554.s008.docx]

| Cell type | Data availability | Reference | Region | Condition |
| --- | --- | --- | --- | --- |
| Neurons | GSE52564 | (1) | Juvenile and adult human brain - cortical tissue | Obtained from surgeries for treating epilepsy and tumours |
|  | GSE149154 | (2) | Healthy post-mortem brain tissue | Pyramidal neurons and aggregcan-positive interneurons |
|  | N/A | (3) | Dorsal Root Ganglia | Whole sensory ganglia from human brain tissue https://bbs.utdallas.edu/painneurosciencelab/sensoryomics/drgtxome/ |
| Astrocytes | GSE52564 | (1) | Juvenile and adult human brain - cortical tissue | Obtained from surgeries for treating epilepsy and tumours |
|  | N/A | (4) | Cingulate cortex | Patients with Huntington's disease compared to controls with no neurological disease |
| Microglia | GSE52564 | (1) | Juvenile and adult human brain - cortical tissue | Obtained from surgeries for treating epilepsy and tumours |
|  | GSE125050 | (5) | Frontal gyrus | RNA sequencing of AD and control frozen brain tissue |
|  | PRJNA544731 | (6, 7) | Human cortex | Single-nucleus sequencing of multiple sclerosis brain |
|  | GSE99074 | (8) | Cortex | Purified human cortical microglia from aged patients with intact cognition |
|  | GSE97930 | (9) | Visual cortex, frontal cortex, cerebellar hemisphere | Post-mortem adult brain 6 brain samples |
| Oligodendrocytes | GSE52564 | (1) | Juvenile and adult human brain - cortical tissue | Obtained from surgeries for treating epilepsy and tumours |

**Supporting Information S4_Table**

**The antimicrobial peptide cathelicidin is critical for the development of Th17 responses in experimental autoimmune encephalomyelitis**

Katie J Smith^1^, Danielle Minns^1^, Brian J McHugh^1^, Rebecca K. Holloway^2,3^, Richard O’Connor^1^, Anna Williams^3^, Lauren Melrose^1^, Rhoanne McPherson^1^, Veronique E. Miron^2^, Donald J Davidson^1^and Emily Gwyer Findlay^1^

1. Zhang Y, Sloan SA, Clarke LE, Caneda C, Plaza CA, Blumenthal PD, et al. Purification and Characterization of Progenitor and Mature Human Astrocytes Reveals Transcriptional and Functional Differences with Mouse. Neuron. 2016;89(1):37-53.

2. Garst-Orozco J, Malik R, Lanz TA, Weber ML, Xi H, Arion D, et al. GluN2D-mediated excitatory drive onto medial prefrontal cortical PV+ fast-spiking inhibitory interneurons. PLoS One. 2020;15(6):e0233895.

3. Ray P, Torck A, Quigley L, Wangzhou A, Neiman M, Rao C, et al. Comparative transcriptome profiling of the human and mouse dorsal root ganglia: an RNA-seq–based resource for pain and sensory neuroscience research. PAIN. 2018;159(7).

4. Al-Dalahmah O, Sosunov AA, Shaik A, Ofori K, Liu Y, Vonsattel JP, et al. Single-nucleus RNA-seq identifies Huntington disease astrocyte states. Acta Neuropathologica Communications. 2020;8(1):19.

5. Srinivasan K, Friedman BA, Larson JL, Lauffer BE, Goldstein LD, Appling LL, et al. Untangling the brain's neuroinflammatory and neurodegenerative transcriptional responses. Nat Commun. 2016;7:11295.

6. Wheeler MA, Clark IC, Tjon EC, Li Z, Zandee SEJ, Couturier CP, et al. MAFG-driven astrocytes promote CNS inflammation. Nature. 2020;578(7796):593-9.

7. Schirmer L, Velmeshev D, Holmqvist S, Kaufmann M, Werneburg S, Jung D, et al. Neuronal vulnerability and multilineage diversity in multiple sclerosis. Nature. 2019;573(7772):75-82.

8. Galatro TF, Holtman IR, Lerario AM, Vainchtein ID, Brouwer N, Sola PR, et al. Transcriptomic analysis of purified human cortical microglia reveals age-associated changes. Nat Neurosci. 2017;20(8):1162-71.

9. Lake BB, Chen S, Sos BC, Fan J, Kaeser GE, Yung YC, et al. Integrative single-cell analysis of transcriptional and epigenetic states in the human adult brain. Nat Biotechnol. 2018;36(1):70-80.
